# Successful behavior change in obesity interventions in adults: a systematic review of self-regulation mediators

**DOI:** 10.1186/s12916-015-0323-6

**Published:** 2015-04-16

**Authors:** Pedro J Teixeira, Eliana V Carraça, Marta M Marques, Harry Rutter, Jean-Michel Oppert, Ilse De Bourdeaudhuij, Jeroen Lakerveld, Johannes Brug

**Affiliations:** 1 grid.9983.b0000000121814263Interdisciplinary Centre for the Study of Human Performance (CIPER), Faculty of Human Kinetics, University of Lisbon, Lisbon, Portugal; 2 grid.8991.9000000040425469XEuropean Centre on Health of Societies in Transition, London School of Hygiene and Tropical Medicine, London, UK; 3 grid.5805.80000000119553500Department of Nutrition Pitié-Salpetrière (AP-HP), Université Pierre et Marie Curie-Paris 6, Paris, France; 4 UREN (Nutritional Epidemiology Research Unit), Université Paris 13, Sorbonne Paris Cité, Inserm (U557), Inra (U1125), Cnam, F-93017, Bobigny, France; 5 grid.5342.00000000120697798Department of Movement and Sport Sciences, Ghent University, Ghent, Belgium; 6 grid.16872.3a000000040435165XDepartment of General Practice and Elderly Care, The EMGO Institute for Health and Care Research, VU University Medical Center, Amsterdam, the Netherlands; 7 grid.16872.3a000000040435165XDepartment of Epidemiology and Biostatistics, The EMGO Institute for Health and Care Research, VU University Medical Center, Amsterdam, the Netherlands

**Keywords:** Lifestyle interventions, Obesity, Self-regulation, Weight control, Physical activity, Dietary intake, Maintenance, Behavior change, Mediation analysis

## Abstract

**Background:**

Relapse is high in lifestyle obesity interventions involving behavior and weight change. Identifying mediators of successful outcomes in these interventions is critical to improve effectiveness and to guide approaches to obesity treatment, including resource allocation. This article reviews the most consistent self-regulation mediators of medium- and long-term weight control, physical activity, and dietary intake in clinical and community behavior change interventions targeting overweight/obese adults.

**Methods:**

A comprehensive search of peer-reviewed articles, published since 2000, was conducted on electronic databases (for example, MEDLINE) and journal reference lists. Experimental studies were eligible if they reported intervention effects on hypothesized mediators (self-regulatory and psychological mechanisms) and the association between these and the outcomes of interest (weight change, physical activity, and dietary intake). Quality and content of selected studies were analyzed and findings summarized. Studies with formal mediation analyses were reported separately.

**Results:**

Thirty-five studies were included testing 42 putative mediators. Ten studies used formal mediation analyses. Twenty-eight studies were randomized controlled trials, mainly aiming at weight loss or maintenance (n = 21). Targeted participants were obese (n = 26) or overweight individuals, aged between 25 to 44 years (n = 23), and 13 studies targeted women only. In terms of study quality, 13 trials were rated as “strong”, 15 as “moderate”, and 7 studies as “weak”. In addition, methodological quality of formal mediation analyses was “medium”. Identified mediators for medium-/long-term weight control were higher levels of autonomous motivation, self-efficacy/barriers, self-regulation skills (such as self-monitoring), flexible eating restraint, and positive body image. For physical activity, significant putative mediators were high autonomous motivation, self-efficacy, and use of self-regulation skills. For dietary intake, the evidence was much less clear, and no consistent mediators were identified.

**Conclusions:**

This is the first systematic review of mediational psychological mechanisms of successful outcomes in obesity-related lifestyle change interventions. Despite limited evidence, higher autonomous motivation, self-efficacy, and self-regulation skills emerged as the best predictors of beneficial weight and physical activity outcomes; for weight control, positive body image and flexible eating restraint may additionally improve outcomes. These variables represent possible targets for future lifestyle interventions in overweight/obese populations.

**Electronic supplementary material:**

The online version of this article (doi:10.1186/s12916-015-0323-6) contains supplementary material, which is available to authorized users.

## Background

Lifestyle treatment interventions for obesity typically target changes in diet and physical activity through strategies like setting adequate goals and enhancing patients’ motivation, changing their beliefs and expectations, and providing guidance in the use of a variety of self-regulation skills (such as self-monitoring), all of which are thought to influence behavior change and maintenance [1-4]. A wide variety of health behavior change theories has been employed to provide conceptual organization of these determinants, including social cognitive theories such as the theory of planned behavior [5], theories of motivation such as self-determination theory [6], theories distinguishing between motivational and post-motivational or volitional phases [7] such as the health action process approach (HAPA) [8], and self-regulation models such as control theory [9]. Since all these theories address the *regulation* of a person’s behavior in the service of some goal or desired outcome, through intrapersonal factors, in this paper we broadly refer to intervening variables in this process as *self-regulation factors*.

Behavior modification in general, and “comprehensive lifestyle interventions” in particular [10] are currently the first recommended step in obesity management. However, so far, randomized controlled trials evaluating the effectiveness of programs that target lifestyle behavior have shown mixed effects and, if effective, they have generally resulted in only small changes in target behaviors [11-15]. In addition, the evidence shows that relatively little if any weight loss accomplished in treatment programs is maintained over the long term [16]. Furthermore, few studies have analyzed why, or by which mechanisms, interventions are successful for some individuals and not for others. Clearly, there is a need for research that identifies causal predictors of long-term weight control, including successful weight loss and maintenance [17].

Despite the limited success of available interventions in reversing the current trends in obesity prevalence, approaches focusing on individual behavior change remain an important topic of interest in obesity research. Several reasons justify this assertion. First, these interventions typically focus on behaviors (for example, diet and physical activity), which have widespread consequences for health, with or without weight loss. Second, if and when individuals are able to successfully self-regulate their behaviors, these effects tend to be sustainable, which is essential for having a lasting impact on health; moreover, this successful self-regulation may also “transfer” to, and help change, other health behaviors [18]. Third, although some interventions targeting individuals may be ineffective on their own, they might be able to contribute to the effectiveness of strategies that integrate multiple levels (that is, strategies that include individual-level and environmental-level approaches) [19]. Finally, the potential for dissemination of individual-level intervention approaches is considerable, given that a sizable number of overweight and obese persons will seek professional help at some point in their lives. Consequently, improving the efficacy of such interventions has substantial clinical as well as public health relevance.

One recent development in studies testing lifestyle interventions for obesity is their ability to identify the mechanisms or processes by which interventions induce meaningful and lasting change in their (most successful) participants. These mechanisms can generally be named *predictors* (or *determinants*) of success, and some studies have gone one step further to evaluate the extent to which they may be causal *mediators* of intervention effects. Testing of mediation, using appropriate methods, is a critical step in this field; it provides the strongest possible inference for the identification of elements in interventions which are causally “responsible” for achieving desired outcomes [20].

Success and failure in the self-regulation of health behaviors involve multiple psychological and behavioral aspects. The aim of this review was to identify and summarize psychological self-regulation mediators of successful weight change, or change in energy balance-related behavior (physical activity and diet), in clinical and community behavior change obesity interventions. Because eventual weight regain is frequent after behavior and/or weight change interventions, particular attention was given to studies reporting long-term outcomes, that is, one year or more after the beginning of the intervention.

## Methods

This systematic review was conducted in accordance with the Preferred Reporting Items for Systematic Reviews and Meta-Analyses (PRISMA) statement [21].

### Eligibility criteria

Studies were included in this review if they were intervention studies published since 2000 in the English language, used experimental designs, and referred to clinical or community behavior change interventions with overweight/obese adults (≥18 years old) aiming to reduce overweight/obesity. This review was limited to “lifestyle interventions” defined as interventions that promote change in energy balance-related behaviors (such as diet and physical activity, as the outcomes) and self-regulatory factors (such as motivation and self-monitoring, as the potential mediators) relevant for overweight/obesity treatment, typically in settings involving personal contact between interventionists and participants. There were no restrictions with respect to the format and duration of the intervention. To be eligible, studies should also report outcomes assessed at least 6 months after the start of the intervention; include a quantitative assessment of change in weight/BMI, physical activity (for example, self-reported or accelerometer-derived minutes of moderate and vigorous physical activity, daily pedometer steps, attendance to PA sessions), or dietary intake (for example, energy intake, fat intake, fruit and vegetable intake) as well as a quantitative assessment of potential mediators of successful behavior change. We decided not to distinguish predictors of weight loss and predictors of weight loss maintenance, choosing instead to divide the studies according to the length of measurement periods (shorter versus longer than 12 months). While it is possible that predictors of those two processes differ, to appropriately evaluate predictors of weight loss maintenance, we would have to rely on studies of successful weight losers, and preferably including psychological measures before and after the maintenance period. Only one intervention study fit both criteria.

An *a priori* list of mediators was used for study inclusion/exclusion, based on previous work in this area (for example, [2,22]). Only mediators representing individual-level self-regulatory processes were considered (that is, those related to skills, motivation, competence*, coping* mechanisms, beliefs, physical self-perceptions, and eating regulation factors such as disinhibition, restraint, and perceived hunger). Mediators associated with personality factors, social support, and health-related outcomes (such as psychological distress, quality of life, and well-being) were excluded. Finally, eligible studies were required to report the effect of the intervention on hypothesized mediator(s) and the association of the putative mediator with the outcomes of interest.

### Search strategy and study selection

A comprehensive search of peer-reviewed articles published between January 2000 and February 2014 (including online ahead of print publication) was conducted in six electronic databases (Pubmed, MEDLINE, PsycINFO, the Cochrane Library, Web of Knowledge, and SPORTDiscus). The decision to restrict the selection to studies published since 2000 is based on the fact that recent development in studies testing the effectiveness of lifestyle interventions for obesity makes older studies less externally valid. For instance, in 1995, Friedman and Brownell [23] alerted for the need of a “third generation” of obesity treatment studies analyzing causal mechanisms between psychosocial variables and weight change. Despite this, one decade later, it has been observed that very few studies had investigated such mechanisms, and even fewer looked into long-term changes [2].

Searches included various combinations of four sets of terms: i) terms concerning the health condition or population of interest (overweight/obesity); ii) terms concerning the intervention(s)/exposure(s) evaluated (for example, behavior change/lifestyle obesity interventions); iii) terms respecting the outcomes of interest (weight change, physical activity, and dietary intake); iv) terms concerning the predictors/mediators of interest (psychological, self-regulation); and v) terms concerning the type of analyses of relevance (for example, mediation, correlates, predictors). (See Additional file 1 for a search example; complete search strategies can be obtained from the authors). Other sources included manual cross-referencing of bibliographies cited in previous reviews [2,22,24-26] and included studies, as well as manual searches of the content of key scientific journals (*Obesity Reviews; International Journal of Obesity; Obesity (Silver Spring); International Journal of Behavioral Nutrition and Physical Activity; Journal of the American Dietetic Association; Psychology of Sports and Exercise; Health Psychology; Journal of Behavioral Medicine; Preventive Medicine*).

Titles, abstracts, and references of potential articles were reviewed by two authors (EVC, MM) to identify studies that met the eligibility criteria. Duplicate entries were manually removed. Relevant articles were then retrieved for a full read. The same two authors reviewed the full text of potential studies, and decisions to include or exclude studies in the review were made by consensus.

### Data coding and extraction

A data extraction form was developed, informed by the PRISMA statement for reporting systematic reviews [21] and the Cochrane Collaboration’s tool for assessing risk of bias [27]. Data extraction included information about study details (authors, year, country of publication, affiliations, and funding), participants (characteristics, recruitment, setting, attrition, compliance, and blinding), study design and setting, outcomes of interest, mediators/predictors (in/out list), intervention length and characteristics, psychosocial instruments, and statistical analysis, including mediation techniques (a complete coding form can be obtained from the authors). Authors of included studies were contacted when necessary to retrieve missing data in published reports.

Considering that the main focus of this review was the identification of mediators, data extraction was performed separately, starting with the studies formally testing mediation (see Additional file 2), followed by those that reported both the effect of the intervention on hypothesized mediators (*path a*) and the association of the putative mediator with the outcomes of interest (*path b*), but did not test mediation (see Additional file 3). Regarding mediation and specifically in studies with formal mediation tests, researchers could use Baron and Kenny’s approach [28] and check whether the main effects were reduced in the presence of the mediator, or employ more sophisticated techniques to directly test the significance of the indirect effect through the mediator (for instance, by following MacKinnon’s approach [29], and using Preacher and Hayes mediation procedures or structural equation modeling). Additional file 4 presents a detailed description of the mediation analyses procedures and estimates for each study. In the latter (that is, predictor studies), we generally looked at a) whether significant intervention-control differences existed for a given variable (or pre-post change in non-controlled designs); b) whether there was an association between these changes (in intervention group only) and changes in the outcome (weight/PA/diet) in this group. If both were present, results were deemed consistent with mediation.

### Quality assessment

The quality of included studies was assessed using an adapted version of the Quality Assessment Tool for Quantitative Studies, developed by the Effective Public Health Practice Project [30], and recommended for use by the Cochrane Public Health Review Group [27]. The current adaptation was based on recommendations from several authors [31,32], and has been used in a previous systematic review conducted as part of the SPOTLIGHT project [33]. This tool was adapted to allow the evaluation of both experimental and observational studies and contains 19 items, guiding the assessment of eight key methodological domains – 1) study design, 2) blinding, 3) representativeness (selection bias), 4) representativeness (withdrawals/dropouts), 5) confounders, 6) data collection, 7) data analysis, and 8) reporting. Each domain is classified as *Strong* (low risk of bias/high methodological quality), *Moderate*, or *Low* (high risk of bias/low methodological quality) methodological quality. A global rating is determined based on the scores of each component (see Additional file 5 for a full description of the Assessment Tool components and scoring system). Two researchers independently rated each of the eight domains and overall quality (EVC, MM). Discrepancies were resolved by consensus.

For studies employing formal tests of mediation, assessment of methodological quality was complemented with a checklist tool developed specifically for mediation analysis by Lubans, Foster, and Biddle [34], and subsequently adapted by Rhodes and Pfaeffli [35]. This tool includes 11 questions answered with a yes (1) or no (0) format, whose scores are added to generate a global score. High quality is represented by scores between 9 and 11, moderate quality ranges between 5 and 8, and low quality is considered when scores are below 5 (see Additional file 5 for a full description of the Checklist components and scoring system). Methodological quality of the mediation analyses was also rated by two authors (EVC, MM), with conflicting judgments discussed to reach agreement. Inter-rater agreement was good (Cohen’s kappa = 0.78).

### Data synthesis

This review analyzed psychological and self-regulation mediators and predictors of change in body weight or BMI (primary outcome), physical activity, and dietary intake, separately (Note: we will use the term *predictors* when studies did not test for formal mediation, and *mediators* when they did). Intervention effects on the outcomes of interest were included in Additional files 2 and 3. Results were divided according to the length of assessment of the outcomes, into short-term (<12 months from the start of the intervention) and long-term (≥12 months), so that those variables mediating/predicting more sustainable outcomes (the main focus of this review) could be more easily identified. Twelve months has been indicated by an expert panel on obesity as an appropriate threshold between weight loss and the maintenance of the weight lost [10]. In the synthesis of data derived from studies formally testing mediation, only controlled trials were included, to further strengthen inference regarding intervention effects on mediators and outcomes. In the case of prediction studies (not formally testing mediation), we included both controlled and uncontrolled trials, to capitalize on the (relatively) larger number of studies available, which would otherwise be excluded using the more stringent criteria. Table 1 describes the 35 included studies. In Tables 2, 3, 4, 5, 6, and 7, mediation-specific results are discriminated from the general results, provided that the main goal of this review was the identification of self-regulation mediators in behavior change obesity interventions. The overall results (considering multivariate, bivariate/correlational, and mediation analyses) are also presented in each table (Tables 2, 3, 4, 5, 6, and 7). A total of 42 mediators/predictors were identified across outcomes. To facilitate data interpretation, considering the very large number of individual variables, these were grouped together by similarity into categories. Categorization was done through the extraction of information from primary studies on the definition and operationalization of the constructs. The following 12 categories were formed: Self-regulatory skills use, Processes of change, Coping mechanisms, Self-efficacy/barriers, Autonomous motivation, Controlled motivation, Decisional balance, Outcomes expectations/beliefs, Body image/physical self-worth, Cognitive restraint, Eating disinhibition, Perceived hunger. Please refer to Additional file 6 for full details regarding the mediators/predictors identified per outcome.

**Table 1:** **Characteristics of intervention studies examining potential mediators/predictors of weight control, physical activity, and food intake (n = 35)**

**Characteristics**	**Number of studies**	**Characteristics**	**Number of studies**
***Study design (n = 35)***		***Intervention (n = 35)***	
*Trial*		*Aim*	
NCT	6	Weight loss	15
NRCT	1	Weight maintenance	6
RCT	28	Exercise adherence	3
*Arms*		Health promotion	8
1 arm	6	Other	2
2 arms	18	*Theoretical grounds* ^*b*^	
3 arms	8	SCT	23
4 or + arms	3	TTM	5
*Sample size*		SDT	3
<100	2	Other	8
100-199	12	N.R.	4
200-299	11	*Intervention setting*	
≥300	10	University	15
		Hospital/clinic	3
***Participants (n = 35)***		Exercise/fitness club	12
*Gender*		Community	3
Women only	13	Web	1
Both genders	22	Other	1
*Mean age, years*		*Intervention length*	
25-44	23	<6 months	6
45-64	11	6-12 months	16
N.R.	1	12-24 months	12
*BMI*		≥24 months	1
25-29.9	2	*Post-intervention follow-up (n = 11)*	
30-34.9	14	<6 months	1
35-39.9	4	6-12 months	5
≥40	8	12-24 months	4
N.R.	7	≥24 months	1
**Characteristics**	**Number of studies**	**Characteristics**	**Number of studies**
***Mediation analysis (n = 10)***		***Outcomes***	
*Type of test*		*Weight change (n = 26)*	
Regression-based	2	Short-term (<12 months)	9
P and H/bootstrap	4	Long-term (≥12 months)	17
SEM/bootstrap	4		
*Mediation approach (and criteria)* ^*a*^		*Physical activity (n = 19)*	
Baron and Kenny	3	Short-term (<12 months)	11
MacKinnon et al.	7	Long-term (≥12 months)	8
Shrout and Bolger	2	Self-reported (MVPA)	15
MacArthur	1	Self-reported (LPA)	2
		Self-reported (Energy Exp.)	1
***Quality assessment score***		Objective^c, d^	4
*Quality score (EPHPP tool; n = 35)*			
Weak	7	*Food/energy intake (n = 11)* ^*e*^	
Moderate	15	Short-term (<12 months)	7
Strong	13	Long-term (≥12 months)	4
*Quality score (Rhodes tool; n = 10)*		Total energy intake	3
Low	0	Fat/saturated fat	6
Moderate	10	Fruit/vegetable	7
High	0	Other (CH; protein)	1

Notes: NCT, non-controlled trial; NRCT, non-randomized controlled trial (for example, a study comparing two interventions, but without a real control group); RCT, randomized controlled trial (Note: two studies referred to post hoc analyses of existing RCTs, for outcomes that were not planned originally); N.R., not reported; P and H, Preacher and Hayes mediation procedures; SEM, structural equation modeling; EPHPP, Effective Public Health Practice Project; SCT, socio-cognitive theory; TTM, transtheoretical model; SDT, self-determination theory; MVPA, moderate and vigorous physical activity; LPA, lifestyle physical activity; Energy Exp., energy expenditure. ^a^Two studies used more than one mediation approach. ^b^Six studies used more than one theoretical framework. ^c^Objective assessments included accelerometry (n = 1), pedometry (n = 1), and actual attendance to a fitness club (n = 2). ^d^Two of the studies also assessed self-reported physical activity. ^e^Three studies assessed more than one outcome.

**Table 2:** **Mediators/predictors of short-term weight control (<12 months)**

**Putative mediators (categories)**	**Formal mediation analyses**			**All analyses**		
	**Number of studies**	**Times tested**	**Effect, %**	**Number of studies**	**Times tested**	**Effect, %**
↑ Self-regulation skill use				6	12	92^a^
↑ Self-efficacy/barriers				6	9	67*^b^
↑ Body image/physical self-worth				2	6	67*
↓ Eating disinhibition				1	4	75*^c^
↑ Cognitive restraint				1	3	33*^a^
↑ Processes of change				1	2	50*
↑ Decisional balance (pros/cons)				1	1	100*
↓ Perceived hunger				1	1	100*^c^
↑ Positive outcome expectations				1	1	0

Notes: Blank spaces correspond to variables that were not tested using formal mediation analyses. *Times tested* refers to the number of times a variable was analyzed; *Effect, %* refers to the number of times an effect was found and is expressed as percentage. Since mediation analyses were not conducted, results are organized according to the number of times each variable was tested in the overall analyses, depicted in the fifth column, in descent order. * ≥ 50% of these effects are based on correlational analyses. ^a^Tested twice in weak quality studies. ^b^Tested three times in weak quality studies. ^c^Tested in a weak quality study. [↑] means that higher value of (variable) was associated with improved outcomes; [↓] means "lower value of (variable)" was associated with improved outcomes.

**Table 3:** **Mediators/predictors of medium/long-term weight control (≥12 months)**

**Putative mediators (categories)**	**Formal mediation analyses**			**All analyses**		
	**Number of studies**	**Times tested**	**Effect, %**	**Number of studies**	**Times tested**	**Effect, %**
↓ Controlled motivation for PA	1	4	0	1	8	0
↑ Self-regulation skill use	3	3	75	6	6	83^a^
↑ Body image/self-worth	2	3	100	4	34	62*
↑ Self-efficacy/barriers	2	3	67	6	28	68*
↑ Autonomous motivation for PA	1	2	100	2	8	100*
↑ Flexible restraint	1	2	100	2	5	60
↑ Positive outcome expectations/beliefs	1	2	0	3	6	50
↓ Eating disinhibition	1	1	100	3	16	38*
↑ Cognitive restraint (total)				4	8	50*
↓ Perceived hunger				3	5	20*
↑ Coping mechanisms				1	2	0

Notes: Blank spaces correspond to variables that were not tested using formal mediation analyses. *Times tested* refers to the number of times a variable was analyzed; *Effect, %* refers to the number of times an effect was found and is expressed as percentage. Results are organized according to the number of times each variable was tested in mediation analyses, depicted in the second column, in descent order. For variables not tested in mediation analyses, the fifth column (times tested in overall analyses) should be taken as the reference. * ≥ 50% of these effects are based on correlational analyses. ^a^Tested once in a weak quality study. [↑] means that higher value of (variable) was associated with improved outcomes; [↓] means "lower value of (variable)" was associated with improved outcomes.

**Table 4:** **Mediators/predictors of short-term physical activity (<12 months)**

**Putative mediators (categories)**	**Formal mediation analyses**			**All analyses**		
	**Number of studies**	**Times tested**	**Effect, %**	**Number of studies**	**Times tested**	**Effect, %**
↑ Body image/physical self-worth	1	2	50	3	6	67*^a^
↑ Self-efficacy/barriers	1	1	0	10	15	67*
↑ Self-regulation skill usage				7	13	85*
↑ Motivational readiness				1	2	50
↑ Processes of change				1	2	50*
↑ Decisional balance (pros/cons)				1	1	100*
↑ Positive outcome expectations/beliefs				1	1	0

Notes: Blank spaces correspond to variables that were not tested in formal mediation analyses. *Times tested* refers to the number of times a variable was analyzed; *Effect, %* refers to the number of times an effect was found and is expressed as percentage. Results are organized according to the number of times each variable was tested in mediation analyses, depicted in the second column, in descent order. For variables not tested in mediation analyses, the fifth column (times tested in overall analyses) should be taken as the reference. * ≥ 50% of these effects are based on correlational analyses. ^a^Tested once in a weak quality study. [↑] means that higher value of (variable) was associated with improved outcomes.

**Table 5:** **Mediators/predictors of medium/long-term physical activity (≥12 months)**

**Putative mediators (categories)**	**Formal mediation analyses**			**All analyses**		
	**Number of studies**	**Times tested**	**Effect, %**	**Number of studies**	**Times tested**	**Effect, %**
↓ Controlled motivation for PA	2	8	0	2	12	25*
↑ Autonomous motivation for PA	2	6	83	2	14	93*
↑ Self-efficacy/barriers	4	6	67^a^	6	12	75^b^
↑ Positive outcome expectations/beliefs	2	3	0	3	3	0
↑ Self-regulation skill usage	2	2	50^a^	3	3	67^b^
↑ Coping mechanisms	1	2	0	1	2	0
↑ Decisional balance (pros/cons)				1	2	0
↑ Cognitive restraint				1	3	0
↑ Processes of change				1	2	0

Notes: Blank spaces correspond to variables that were not tested in formal mediation analyses. *Times tested* refers to the number of times a variable was analyzed; *Effect, %* refers to the number of times an effect was found and is expressed as percentage. Results are organized according to the number of times each variable was tested in mediation analyses, depicted in the second column, in descent order. For variables not tested in mediation analyses, the fifth column (times tested in overall analyses) should be taken as the reference. * ≥ 50% of these effects are based on correlational analyses. ^a^Tested once in a weak quality study. ^b^Tested twice in weak quality studies. [↑] means that higher value of (variable) was associated with improved outcomes; [↓] means "lower value of (variable)" was associated with improved outcomes.

**Table 6:** **Mediators/predictors of short-term dietary intake (<12 months)**

**Putative mediators (categories)**	**Formal mediation analyses**			**All analyses**		
	**Number of studies**	**Times tested**	**Effect, %**	**Number of studies**	**Times tested**	**Effect, %**
↑ Self-efficacy/barriers				6	12	75*
↑ Self-regulation skill usage				5	12	75*
↑ Motivational readiness				1	4	0

Notes: Blank spaces correspond to variables that were not tested in formal mediation analyses. *Times tested* refers to the number of times a variable was analyzed; *Effect, %* refers to the number of times an effect was found and is expressed as percentage. Since mediation analyses were not conducted, results are organized according to the number of times each variable was tested in the overall analyses, depicted in the fifth column, in descent order. * ≥ 50% of these effects are based on correlational analyses. [↑] means that higher value of (variable) was associated with improved outcomes.

**Table 7:** **Mediators/predictors of medium/long-term dietary intake (≥12 months)**

**Putative mediators (categories)**	**Formal mediation analyses**			**All analyses**		
	**Number of studies**	**Times tested**	**Effect, %**	**Number of studies**	**Times tested**	**Effect, %**
↑ Self-efficacy/barriers	1	2	50	3	8	25
↑ Coping mechanisms	1	2	0	2	4	0
↑ Positive outcome expectations/beliefs	1	1	0	2	2	0
↑ Processes of change				1	8	0
↑ Cognitive restraint				1	6	0
↑ Decisional balance (pros/cons)				1	4	0
↑ Self-regulation skill usage				2	2	100^a^

Notes: Blank spaces correspond to variables that were not tested in formal mediation analyses. *Times tested* refers to the number of times a variable was analyzed; *Effect, %* refers to the number of times an effect was found and is expressed as percentage. Results are organized according to the number of times each variable was tested in mediation analyses, depicted in the second column, in descent order. For variables not tested in mediation analyses, the fifth column (times tested in overall analyses) should be taken as the reference. ^a^Tested twice in weak quality studies. [↑] means that higher value of (variable) was associated with improved outcomes.

Finally, Tables 2, 3, 4, 5, 6, and 7 show, separately for each mediator/predictor, the number of studies that have analyzed it, the number of times it was tested (some of them within the same study), and the number of times a significant effect was found. Results are presented for mediation-specific results and for the overall results.

## Results

### Study selection

**Figure 1:**
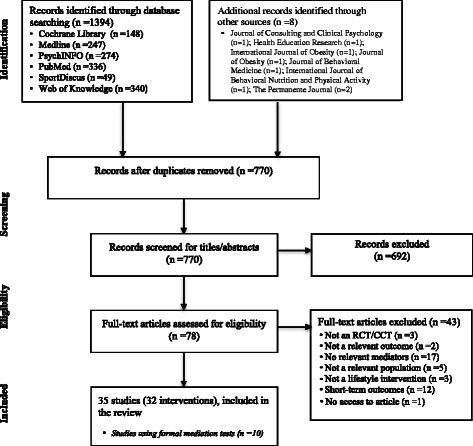
Flow diagram of studies.

The literature search yielded a total of 1,394 potentially relevant records. Eight additional articles that were identified through manual searches and cross-referencing were added, bringing the total number of potential articles to 1,404. Of these, 770 abstracts were assessed for eligibility (634 duplicates removed). After the initial screening of titles and abstracts, 692 articles were excluded (Figure 1). Some articles were excluded for multiple reasons. Thirty-five articles describing 32 unique lifestyle interventions met the eligibility criteria and were therefore included [36-70]. Papers reporting on the same intervention are identified in Additional files 2 and 3.

### Study characteristics

The characteristics of included studies are summarized in Table 1 (for further details, see Additional files 2 and 3). Most studies (n = 28) were randomized controlled trials, mainly aiming at weight loss or weight loss maintenance (n = 21). Most interventions took place in university (n = 15) or fitness club settings (n = 12), and most lasted more than 6 months (n = 29). However, only 11 trials included a follow-up assessment period and, of these, more than half were shorter than 12 months. Most studies were based on, or at least informed by, one or more theories of behavior change; the most frequent being social cognitive theory (n = 23), the transtheoretical model (n = 5), and self-determination theory (n = 3). Eight interventions were grounded in other theories, including group dynamics theory, problem solving model, theory of planned behavior, health belief model, and self-regulation theory. Four studies did not report using any theoretical framework. Samples were mostly composed of obese individuals (n = 26), aged between 25 to 44 years old (n = 23), and 13 studies targeted women only.

Twenty-six studies evaluated mediators/predictors of weight change, of which 17 reported medium/long-term outcomes; 19 studies evaluated mediators/predictors of physical activity, with 8 of them reporting medium/long-term outcomes; finally, 11 studies investigated dietary intake as the outcome measure, 4 of them in the medium/long-term. Weight-related measurements were performed with calibrated digital scales, and weight changes were expressed in weight change percent from baseline (n = 9), in kilograms (n = 10), as residualized scores regressed on the baseline scores (n = 6), or as BMI changes (n = 3). Regarding physical activity, objective measures were employed in 4 studies (for example, accelerometry, pedometry) and self-reported instruments in 17 studies; of these, 6 used the Seven-Day Physical Activity Recall [71] and 6 studies used the Godin Leisure-Time Exercise Questionnaire [72]. Dietary and caloric intake, indirectly assessed through the number of servings, was collected with the Food Intake Questionnaire in most studies (n = 5), followed by the Three-Day Food Records in most of the studies (n = 3), and the Block Food-Frequency Questionnaire (n = 1).

### Quality assessment

The overall results of the quality assessment can be found in Table 1 and the total quality score for each study in Additional files 2 and 3 (for a detailed classification of each item and study see Additional file 7). Regarding the overall methodological quality of the studies, 13 studies were rated as ”strong”, 15 were classified as ”moderate”, and 7 were rated as ”weak”. All included studies scored strong on the *Study design*, as they were experimental. Thirteen studies were rated as weak regarding *Blinding* of participants (during recruitment) and outcome assessors, 13 were rated as moderate, 8 as strong, and 1 did not receive a rating, as it was a non-randomized trial. All studies except two (one scored weak and the other scored strong) scored moderate regarding *Representativeness* (selection bias). Regarding reporting of *Withdrawals and dropouts*, 5 studies were rated as weak, 16 as moderate, and 14 as strong. Four studies scored weak in the adjustment of analysis for *Confounders*, 10 scored moderate, and 21 strong. In terms of *Data collection* tools, 4 studies were rated as weak as they did not provide information on the validity or reliability of the measures used, 11 were classified as moderate, and 17 as strong. Three studies were not rated as they used a larger dataset for which information on psychometric properties of the measures is already provided. All studies scored strong in the use of *Appropriate statistical analyses*. In terms of *Reporting,* 30 studies were rated as strong, and 5 studies as moderate.

In addition, studies including formal tests of mediation (n = 10) were classified as of moderate (n = 10) quality on the mediation analysis checklist. None of the studies reported conducting pilot studies to test mediation, and in all except two studies, there was no specific information regarding the power of the analysis to detect mediation. In only six studies were the outcomes controlled for baseline values.

Mediators/predictors tested in studies with weak methodological quality are identified in Tables 2, 3, 4, 5, 6, and 7. Overall, there appeared to be no association between the methodological quality of the studies and the results of the mediation analyses. Only 2 out of the 7 studies with a global weak score reported significant results for all mediator/predictors.

### Mediators/predictors of weight control

Of the total number of studies investigating mediators/predictors of weight control (n = 26), 9 looked into *short-term* outcomes (<12 months) [47-49,51,52,54,57,62,70]. Twenty-one variables, grouped into nine categories, were tested as mediators/predictors of short-term weight control (Table 2). None of the studies performed formal tests of mediation. In the overall analyses (in this case, all were multivariate), self-regulation skill use emerged as the most consistent predictor of short-term weight control (consistent with mediation in 92% of the times it was tested [12 times in 6 studies]). Other variables that appear promising as mediators of short-term weight control were higher self-efficacy (and/or lower perceived barriers) and more positive body image, both consistent with mediation in 67% of the times they were tested (a total of 9 and 6 times, respectively). In the case of self-efficacy, 2 (out of 6) studies presented with low methodological quality. Although lower eating disinhibition also appears to find empirical support in non-formal mediation analyses, these results come from a single, weak quality study, and are correlational in nature. There were no other consistent mediators/predictors of short-term weight control.

Seventeen studies investigated potential mediators/predictors of *long-term* (≥12 months) weight outcomes, the main focus of the review [36,39,40,43-45,55,56,58,59,63-66]. Of these, six were RCTs that included formal tests of mediation [36,39,40,43-45]. Thirty variables, grouped in 12 categories, were tested as potential mediators/predictors (Table 3). The variables with stronger empirical support in formal mediation studies were body image, which was significant in all the times it was tested (3 times), and self-regulation skills, which was identified as a mediator in 67% of the times it was tested (2 times out of 3 studies). Self-efficacy was a significant mediator in 2 of the 3 times it was tested. For autonomous motivation and flexible eating restraint, results appear promising but derive from a single study in each case. Results observed in non-mediation analyses were consistent with the most stringent analyses, especially those concerning self-regulation skill use, autonomous motivation, and self-efficacy. For self-regulation skill use, significant effects were found in 83% of the 6 times it was tested, and every time in multivariate analyses. For autonomous motivation, results were consistent with mediation in all cases, but they originate from only two studies. On the other hand, empirical support from non-mediation analyses for other variables like body image and self-efficacy appears comparatively weaker and correlation-based; yet, the number of times each of these variables was tested is substantially higher (34 and 28 times, respectively). Eating disinhibition, which appeared to be an additional predictor in the short-term, does not seem to be consistent in the long-term provided that it was significant only in 38% of the 16 times it was tested. There were no other consistent mediators/predictors of long-term weight control.

### Mediators/predictors of physical activity

Of the total number of studies investigating mediators/predictors of physical activity (n = 19), 11 looked into *short-term* outcomes (less than 6 months beyond the start of the intervention) [37,46,51-53,60,61,67-70]. Of these, only one formally tested mediation [37]. Fourteen variables, grouped in seven categories, were tested as mediators/predictors of short-term weight control (Table 4). Regarding mediation-specific results, body image emerged as a significant mediator only in one of the two times it was tested. In non-mediation studies, stronger empirical support was found for self-regulation skill use, which was significant in 11 of the 13 times it was tested (corresponding to 7 different studies). Body image and self-efficacy appear to be promising as mediators of short-term physical activity, showing significant results in 4 (out of 6) and 10 (out of 15) times they were tested, respectively. No other consistent mediators/predictors of short-term physical activity were identified.

Eight studies analyzed mediators/predictors of *long-term* physical activity [36,38,41-43,50,64,65], of which five used formal tests of mediation [36,38,41-43]. Twenty-three variables, grouped in nine categories, were tested as predictors (Table 5). The main predictors of long-term physical activity were autonomous motivation and self-efficacy, considering both mediation-specific analyses and the overall analyses. For autonomous motivation, results from two studies showed that mediation analyses were significant in 83% of the times and overall analyses showed consistency with mediation in 93% of the times (out of 14). For self-efficacy, results originated from 6 different studies. Mediation analyses were significant in 67% of the times self-efficacy was tested (6 times); and in the overall analyses, results were consistent with mediation in 75% of the times (out of 12). Controlled motivation was also consistently unrelated with physical activity outcomes, independent of the type of analyses performed. Finally, self-regulation skill use appears to mediate long-term physical activity in one out of two (formal mediation) and two out of the three (all analyses) times tested, but these results derive from two studies with low methodological quality.

### Mediators/predictors of dietary intake

Of the total number of studies (n = 11) investigating mediators/predictors of dietary intake, seven looked into short-term outcomes [46,53,61,67-70] and four into long-term outcomes [41,50,64,65]. Only one study formally tested mediation [41]. Seven variables (grouped in three categories) were tested as mediators/predictors of short-term dietary intake, while 12 variables (grouped in seven categories) were tested in the long-term (Tables 6 and 7). Self-efficacy/barriers and self-regulation skill use appear promising as mediators of dietary intake in the short-term, both showing results consistent with mediation in 75% of the times they were tested (12 times out of 6 studies for self-efficacy, and 12 times out of 5 studies for self-regulation skills). No consistent mediators/predictors were identified in the longer time frame. Yet, self-efficacy was consistently unrelated with long-term dietary intake, looking less promising as a mediator (results were consistent with mediation only in 2 of the 8 times it was tested).

## Discussion

This review sought to identify the most consistent individual-level mediators of weight change, physical activity, and obesity-related dietary variables, in the context of lifestyle obesity interventions aimed at overweight and obese adults. These mediators or predictors of intervention effects were assessed by self-report, and are thought to represent psychological *mechanisms* or *processes* by which interventions affect body weight, through changes in energy-balance related behaviors. Note that this review did not focus on the efficacy of the interventions’ main effects *per se.* However, mediation mechanisms can be evaluated even in the absence of main significant effects of interventions, particularly in controlled trials [20].

Special emphasis was given to variables tested as formal mediators of changes in the outcomes of interest, as this provides the best possible inferences regarding causal determinants of behavior change [73]; to the extent a consistent mediator is identified, it can more confidently be targeted in future interventions of comparable characteristics. Moreover, because it is unlikely that any single factor (self-regulatory or otherwise) by itself will explain a large share of variance of change in complex behaviors such as physical activity and diet (as a result of an intervention), the identification of groups of significant predictors, which can be then discussed in the context of current theories of behavior change, can additionally contribute to understanding the role of theory in health behavior change [74,75].

As in many systematic reviews of behavior change interventions, the diversity of studies available - reflected on a similarly diverse set of independent (and dependent) variables, study designs, measurement methods, populations represented, and so forth - is a substantial limitation. In the present review, the large number of predictors per study, combined with substantial heterogeneity in study length, type and format of interventions (for example, web-based, face to face, group-based), and assessments employed for each variable made the task at hand especially difficult. In this scenario, the fact that several variables were identified as predictors or, in some cases, actual mediators of intervention-related change in weight control and physical activity is encouraging. Specifically, the present review shows that positive changes in body image, in autonomous motivation for physical activity, in self-efficacy (and fewer perceived barriers), and in the use of self-regulation skills (such as self-monitoring) are promising aspects that may *explain* the variability of results in current lifestyle obesity treatment interventions. Increases in flexible restraint could also be in this group with respect to weight outcomes, but with lower inference. Therefore, these are currently the best evidence-based candidates to target in future individual-level, real-world interventions in this domain.

Some qualifications to the previous conclusions are of note. First, for short-term results, formal tests of mediation were only reported for one of the outcomes of interest (physical activity) and taking into account only two mediators (body image/self-worth and self-efficacy/barriers). Second, there are currently too few studies using dietary variables as outcomes to allow us to draw meaningful conclusions, and only one study tested formal mediation for both time frames. Third, the external validity of some of the reported findings, such as regarding self-regulation skills and autonomous motivation, may be limited, because these findings were derived from few studies conducted by a small number of research groups, using similar study designs.

Body image appeared important as a mechanism leading to change in body weight in several studies. Body image is a multidimensional concept [76] that depicts attitudes, perceptions, and in some cases behaviors associated with mental representations of one’s body (or some of its parts) [77,78]. Poor body image often reflects a high level of concern with body weight or shape, what is known as dysfunctional investment in body image, when body esteem occupies an excessive role as a determinant of overall self-esteem [79]. Previous reviews [2,22] have identified poor body image as a predictor of less success at body weight loss (or, conversely, better body image as a positive factor in obesity treatment interventions). Potential reasons for this association range from excessive psychological pressure leading to more rigid and inconsistent eating regulation [80-82] - poor body image being associated with a history of failed attempts and thus being a marker for other physiological, psychological, or socio-environmental risk factors for weight gain/regain [83,84] - to motivational factors in which external pressures and goals predominate but tend not to produce behavior change in consistent or healthy ways (for example, wanting to be thin for reasons related to social comparison and perceived desirability) [85-87].

Autonomous motivation, a concept derived from self-determination theory (SDT, [88]), generally indicates the degree to which individuals self-endorse, feel that they have a choice about, and attribute deeply reflected value to a certain behavior. In contrast with the most common quantitative view of motivation (how much?), the level of autonomy represents a qualitative analysis of people’s psychological energy to act, which is perceived as internal (reflecting a sense of “ownership” over the behavior). Autonomous motivation is often associated with goals such as pursuing positive personal challenges, attaining/preserving health and well-being, social affiliation, personal development, and self-expression [89]. Additionally, because self-determined, well-internalized behaviors are associated with the satisfaction of the needs for autonomy, competence, and relatedness - and with the feelings of internal coherence and well-being that are thought to emerge from those experiences - this provides an explanation for the behavior to be pursued consistently [89]. A recent meta-analysis [90] and other reviews provide empirical support for both the SDT motivation model and the association of autonomous motivation with health behavior change in different areas [91].

Self-efficacy and perceived barriers are common variables in several theoretical frameworks concerned with health behaviors [92,93]. Self-efficacy measures one’s confidence to successfully implement a course of action by successfully organizing internal and external resources [94]. Although efficacy can be assessed towards other aspects of behavior regulation, it is commonly conceptualized and assessed as “barriers efficacy” or confidence to overcome internal or external obstacles that may stand in the way of one’s actions. Indeed, the correlation between self-efficacy and perceived barriers is usually high [56] (which explains our decision to group these variables in the same category). Although conceptual differences exist, self-efficacy is often equated to the concept of perceived behavior control (from the theory of planned behavior) or perceived competence (as used in self-determination theory). In practical and simple terms, enhancing confidence and competence about a given health behavior appears to be helpful in overcoming barriers - namely in initial stages of adoption - and is often a first step to increase and improve motivation for change.

Flexible eating restraint involves regulating one’s food intake so that no particular behavior is forbidden and thus subject to rigid control and scrutiny [95]. Flexible restraint is generally associated with less internal pressure to diet and a more gradual understanding of the diet’s impact on energy balance. It stands in opposition to rigid restraint [96]. Although, in the past, cognitive restraint was measured as a unified concept, its separation into flexible and rigid dimensions is increasingly frequent in obesity studies and has proven useful in understanding diet and weight regulation, particularly in the long-term. For example, we found that flexible, but not rigid or total restraint, mediated 24-month weight loss in overweight women [39] and, in the present review, results for the total restraint scale and the flexible scale also differed, as in other studies [97]. More broadly, psychological flexibility appears to predict health and psychological well-being [97], is thought to reflect more committed, values-based goal pursuit [98,99] and is considered a hallmark of self-determination [89], factors which may help explain successful health behavior self-regulation.

Finally, the use of certain self-regulation skills, for instance, monitoring weight, diet, and activity, as well as employing goal setting and planning techniques, was also identified as a relatively consistent predictor of successful outcomes, most especially in the shorter-term analyses. In brief, some of these skills may be important for people to ultimately act on their positive intentions. Sometimes associated with self-regulation theories (*cf*. [100]) these variables are more skill-based (in some cases, they are discrete behaviors in themselves) and somewhat different than the previous set of predictors, more intrapsychic. Notably, recent behavior change models focusing on the “intention-behavior gap” (see, for example, [7,101,102]) make the distinction between motivational and implementation phases (sometimes referred to as “volitional” or “post-motivational”), with self-regulation skills reviewed in the present study falling in the latter phase [103]. Results from the present review suggest that some combination of motivational and implementation factors is important. Although this needs confirmation, there is some indication that the latter may be especially useful in early stages of behavioral adoption, whereas motivational factors may be operative along the entire continuum from adoption to maintenance, as highlighted recently in a separate study [104].

In looking at the collective findings from the present analysis, the temptation to interpret them in an integrative way is unavoidable. In principle, there should be “a logic” as to why this set of predictors emerged and not a different one, even considering the intrinsic limitations of the available data (see below). In this exercise, we are informed by our own research, for instance, linking improved eating regulation, including flexible eating, with improved body image [105] and with exercise autonomous motivation [18] and also by other studies. For example, recently, in a large dataset of women in New Zealand, autonomous motivation for eating was associated with less binge eating and slower speed of eating (and a much healthier diet), indicative of improved eating self-regulation [106]. The literature looking at relations between body image and eating behavior is also fertile in suggesting a close association between improved body image, improved eating regulation, and better weight control (see, for example, [55,87]). In this respect, an attempt was recently made to provide an integrative view of eating regulation and weight maintenance, which also includes an explanation for the etiology and role of body image concerns and disordered eating, while considering motivational and self-regulatory aspects [80]. It links goals (such as appearance versus health focus) and the predominant approach to eating regulation (such as rigid versus flexible restraint; focus on quality versus quantity) with the satisfaction of the needs for competence, autonomy, and relatedness, resulting in more or less adaptive diet and weight regulation (see Figures one and two in [80] for more details). The evidence from other recent systematic reviews and meta-analyses, showing that more autonomous forms of health behavior regulation, in physical activity [91], weight control [2], and in health more generally [90] are predictive of better adherence and improved outcomes, is also consistent with the relationships found in the present study.

While some limitations of the present work have been presented above, others need to be considered when interpreting the findings of this review. The large heterogeneity in the study-specific mediation methods and estimates reported in the primary studies prevented us from deriving a single comparable estimate for each variable and reporting on the pooled magnitude of mediation effects. This variability, as well as the limited number of studies for each mediator, did not recommend the use of meta-analytical techniques to pool data across studies. In this review, we used a narrative synthesis approach including vote counting of the number of significant mediation effects for a given variable in relation to all tests of mediation available for that variable. Although this method is not as robust as other quantitative approaches to synthesize data, it provides a reasonably good indication of whether that variable can be identified as a formal mediator (or a variable consistent with mediation) of each intervention, for each specific outcome. It should also be considered that in the primary studies included in this review, statistical significance of the mediation effects was typically the parameter used to infer that a given variable mediated the intervention effect.

Some studies were characterized by poor methodological quality, and none of the studies employing formal mediation analysis presented strong methodological quality. Nonetheless, for most mediators we did not find an association between the methodological quality of the studies and the direction/strength of the effects reported. As exceptions, we did find that in the analyses in which eating disinhibition had consistent significant effects, this was tested mostly in studies of poor quality. A similar result was found for self-regulation skills for the long-term effects in physical activity and dietary intake. Future reviews would benefit from sensitivity analyses. The diversity of outcome measures, especially for physical activity, is also a limitation, as different types of physical activity may be predicted by different factors. The fact that the coding of study characteristics was based on the description provided in the articles is also limiting, given that in many cases these descriptions did not provide enough information regarding mediation analysis, which measures were used, or the content of the interventions. Future studies should provide more detail on the content of the interventions and self-regulation factors addressed to facilitate data interpretation and inference. The choice of the year 2000 to start our search was largely arbitrary and could be seen as a limitation. Finally, the inclusion of non-controlled trials in some of the analyses could be viewed as a limiting factor; on balance, we found this an acceptable compromise (for non-mediation studies only) against the prospect of altogether excluding several studies from this review.

These limitations notwithstanding, this study identified a small number of intervention-related aspects with supporting evidence for an important role played in the difficult path of successful weight control. Since all evidence was derived from intervention studies and independent variables were analyzed as to their mediating position in the behavioral causal chain (although with variable levels of inference), we believe this is a first step leading to their formal inclusion in recommendations for lifestyle programs aiming at weight control. In practical terms, this could mean that strategies or “behavior change techniques” [107] identified as the most effective to specifically change these variables (for example, self-efficacy [108] or autonomous motivation [109]) would be integrated into future interventions in a widespread fashion, and that health professionals would be appropriately trained on how to target them regularly in their practices. It could also mean that bedside instruments (such as brief questionnaires or interview items) would be made available for professionals to quickly assess their patients for these variables (for example, to assess their body image or level of self-regulation skill use [1,110]) and tailor interventions to the most relevant targets for each person. In the area of motivation enhancement, the techniques and instruments used in motivational interviewing [111,112] are a good example of such potential application in medicine and health care.

## Conclusion

In conclusion, based on the scientific literature to date, autonomous motivation, self-efficacy, and self-regulation skill use emerge as the most promising individual-level mediators of positive weight outcomes and increased physical activity. For long-term weight control, promoting a positive body image and flexible eating control may also be important. These aspects represent potential entry points for future lifestyle obesity interventions in adults.

## Additional files

Additional file 1:
**An Example of the Conducted Search (Medline).**
Additional file 2:
**Characteristics of Included Studies With Formal Mediation Analyses.**
Additional file 3:
**Characteristics of Included Studies Without Formal Mediation Analyses.**
Additional file 4:
**Indirect effects’ estimates in studies with formal mediation analysis.**
Additional file 5:
**EPHPP Quality Assessment Tool (adapted by the SPOTLIGHT Consortium).**
Additional file 6:
**Complete results for weight change, physical activity and dietary behaviors.**
Additional file 7:
**Consensus Ratings of Methodological Study Quality.**
