# Supplementary material for: Successful behavior change in obesity interventions in adults: a systematic review of self-regulation mediators
Source: BMC Med. 2015 Apr 16;13:84. doi: 10.1186/s12916-015-0323-6 (PMC4408562; doi:10.1186/s12916-015-0323-6)
Supplement: Additional file 1: — An Example of the Conducted Search (Medline). [file 12916_2015_323_MOESM1_ESM.docx]

**Aditional File 1**

1 (intervention or program or trial or treatment).ab. (2665942)

2 experimental.ab. (532912)

3 randomized.ab. (254524)

4 (randomized controlled trial or controlled clinical trial).pt. (420217)

5 clinical trials as topic.sh. (163042)

6 1 or 2 or 3 or 4 or 5 (3390016)

7 (((weight or BMI or fat or "body fat") and loss) or change or control or management or maintenance).ab. (2548389)

8 (physical activity or exercise or exercise adherence).ab. (172766)

9 (diet or energy intake or caloric intake or calorie intake or dietary).ab. (261079)

10 7 or 8 or 9 (2816617)

11 (obese or overweight or excess weight).tw. (85246)

12 6 and 10 and 11 (18114)

13 limit 12 to "all adult (19 plus years)" (9975)

14 (cognitive* or behav* or psychosocial* or psychological* or motiv* or self-regul*).ab. (900963)

15 13 and 14 (1931)

16 mediat*.ab. (797322)

17 15 and 16 (87)

18 limit 17 to yr="2000 -Current" (81)

19 (predictors or determinants or correlates or moderators).ab. (249722)

20 15 and 19 (168)

21 limit 20 to yr="2000 -Current" (145)

22 18 or 21 (215)

23 22 not 18 (134)

**Figure A.1.** Medline Search Strategy
