# Supplementary material for: Successful behavior change in obesity interventions in adults: a systematic review of self-regulation mediators
Source: BMC Med. 2015 Apr 16;13:84. doi: 10.1186/s12916-015-0323-6 (PMC4408562; doi:10.1186/s12916-015-0323-6)
Supplement: Additional file 2: — Characteristics of Included Studies With Formal Mediation Analyses. [file 12916_2015_323_MOESM2_ESM.docx]

**Additional File 2**

**Table 2.1** Characteristics of included studies with formal mediation analysis

| **Authors** | **Study Design** | **Sample** | **Intervention** | | **Assessment Points** | **Outcomes** | **Mediators** | **Mediation Analysis** | **Study Quality ^1^** |
| --- | --- | --- | --- | --- | --- | --- | --- | --- | --- |
|  |  |  | Aim, rationale, setting/format | Length + Follow-up |  |  |  |  |  |
| Silva et al., 2011[1]*** | RCT; 2 arms | 221 women; Age, 37.6 ± 7.0 yr; BMI, 31.6 ± 4.1 kg/m^2^ | Group-based WL intervention;  Grounded on Self-Determination Theory; University setting | 12 months + 24 months follow-up | 0, 12, 24, 36 months | 24-month MVPA change (7-d PAR): +272 ± 223 min/wk; Cohen’s *d* = 1.22  36-month weight change: -3.9 ± 7.6 % equivalent to ≈ -3.2 kg; *d* = 0.51 | Tested mediators:  12- and 24-month autonomous and controlled (introjected and external) motivation for PA (SRQ-E)  Significant mediators:  Intervention ↑ perceived need support, which ↑12- and 24-month autonomous motivation for PA, which ↑ 24-month **PA** and 36-month **WL.** Correlations between these variables were also significant; 24-month introjected regulation was correlated with PA. | SEM (SmartPLS software); Formal test of mediation, using MacKinnon’s approach  Correlations | Strong  (*EPHPP Tool* - adapted)  Moderate (*Rhodes Tool* - adapted) |
| Annesi et al., 2011[2] | RCT; 2 arms | 137 women; Age, 42.6 ± 10.3 yr; BMI, 37.0 ± 5.7 kg/m^2^ | PA intervention;  Grounded on Social Cognitive Theory;  Exercise/Fitness club setting | 6 months; no follow-up | 0, 6 months | 6-month PA session attendance: 49.3±28.9% | Tested mediators:  Exercise self-efficacy (ExSE), physical self-concept (PSCS), body satisfaction (BAS-MBSRQ)    Significant mediators:  Total indirect effect through all 3 mediators was significant. Intervention-induced ↑ in physical self-concept mediated ↑ in **PA session attendance** | P&H multiple mediation macro; Formal test of mediation, using MacKinnon’s approach | Strong  (*EPHPP Tool* - adapted)  Moderate (*Rhodes Tool* - adapted) |
| Roesch et al, 2010[3] | RCT Post-hoc; 2 arms | 842 (52% men); Age, 42.6 ± 8.42 yr; BMI between 25-40 kg/m^2^ | Web-based health promotion intervention;  Grounded on Social Cognitive Theory and Transtheoretical Model | 12 months; no follow-up | 0, 6, 12 months | 12-month change leisure-time PA (IPAQ): *d* = 0.25 | Tested mediators:  Exercise self-efficacy (ExSE, adapted), behavior change strategies (SSRS scale), decisional balance pros and cons (DBE, adapted)  Significant mediators:  Intervention ↑ behavior change strategies, which ↑ PA. Intervention ↑ exercise self-efficacy, which ↑ **PA**. Behavior change strategies and self-efficacy (but not decisional balance) also predicted PA change in growth models. | SEM (Latent Class Growth Analysis); Formal test of mediation, using MacKinnon’s approach  Regressions | Weak  (*EPHPP Tool* - adapted)  Moderate (*Rhodes Tool* - adapted) |
| **Authors** | **Study Design** | **Sample** | **Intervention** | | **Assessment Points** | **Outcomes** | **Mediators** | **Mediation Analysis** | **Study Quality ^1^** |
|  |  |  | Aim, rationale, setting/format | Length + Follow-up |  |  |  |  |  |
| Teixeira et al., 2010[4] *** | RCT; 2 arms | 225 women; Age, 37.6 ± 7.0 yr; BMI, 31.3 ± 4.1 kg/m^2^ | Group-based WL intervention;  Grounded on Self-Determination Theory; University setting | 12 months + 12 months follow-up | 0, 12, 24 months | 12-month weight loss: -7.3 ± 5.9 % equivalent to ≈ -6 kg; *d* = -1.24  24-month weight loss/maintenance: -5.5 ± 5.0 % equivalent to ≈ 4.5 kg; *d* = -1.10 | Tested mediators:  Cognitive restraint, flexible and rigid restraint, eating disinhibition, hunger (TFEQ); external and emotional eating (DEBQ); eating self-efficacy (WEL), exercise self-efficacy (SEEBS), exercise perceived barriers (EPBS), exercise motivation (IMI), body shape concerns (BSQ); body dissatisfaction (BIA); physical self-worth and body attractiveness (PSPP)  Significant mediators:  ↑ in flexible restraint and ↓ in emotional eating mediated 12-month **weight loss**. ↑ in exercise self-efficacy and flexible restraint, and ↓ in body dissatisfaction mediated 24-month **weight loss/maintenance** (after adjusting for 12-month WL, only self-efficacy remained significant). All restraint and body image variables, emotional eating, PA barriers and self-efficacy, and motivation were correlated to 12-month WL. Physical self-worth, body dissatisfaction, flexible restraint, and exercise self-efficacy were correlated to 24-month WL. | P&H simple mediation; Formal test of mediation, using Shrout & Bolger’s approach, as well as Baron & Kenny’s.  Correlations | Strong  (*EPHPP Tool* - adapted)  Moderate  (*Rhodes Tool* - adapted) |
| Perri et al., 2008[5] | RCT; 3 arms | 234 women; Age 50-75 yr; BMI > 30 kg/m^2^ and weight < 159.1 kg | Extended care (after WL program);  Grounded on Solve Problems Model; Extension Service offices’ setting | 6 months WL program + 6 months extended care (telephone OR face-to-face (group) counseling OR controls) + 6 months follow-up | 6, 18 months | 6-month weight change: Data not reported  18-month weight change: Data not reported | Tested mediators:  Self-monitoring (number of records)  Significant mediators:  ↑ adherence to behavioral self-monitoring strategies mediated the effect of extended care on **weight management** (stronger mediating effect on the telephone counselling group) | Regressions (Baron & Kenny) | Strong  (*EPHPP Tool* - adapted)  Moderate (*Rhodes Tool* - adapted) |
| **Authors** | **Study Design** | **Sample** | **Intervention** | | **Assessment Points** | **Outcomes** | **Mediators** | **Mediation Analysis** | **Study Quality ^1^** |
|  |  |  | Aim, rationale, setting/format | Length + Follow-up |  |  |  |  |  |
| Burke et al., 2010[6]** | RCT; 2 arms | 241 (~52% men); Age 40-70 yr; mean BMI ~30 kg/m^2^ | Intervention to reduce blood pressure; Grounded on Theory of Planned Behavior, Health Belief Model, Transtheoretical Model, Social Cognitive Theory, Decisional Balance; University setting | 4 months + 8 months follow-up | 0, 4, 12 months | MVPA change (7day-PAR): Data not reported.  Change in saturated fat intake, % (3day Food Records): Data not reported. | Tested mediators:  Exercise self-efficacy and diet self-efficacy (PMT-SE), beliefs about benefits of behavior change (created measure), coping mechanisms – consumption and external (WCC-revised), barriers for PA and diet (created measure);  Significant mediators:  12-month reduction in **saturated fat intake** was mediated by ↑ in self-efficacy during the intervention. 12-month change in **PA** was not mediated by these psychosocial variables. | P&H, simple mediation; Formal test of mediation, using MacKinnon’s approach | Moderate  (*EPHPP Tool* - adapted)  Moderate (*Rhodes Tool* - adapted) |
| Silva et al, 2010[7] *** | RCT; 2 arms | 239 women; Age, 37.6 ± 7.1 yr; BMI, 31.5 ± 4.1 kg/m^2^ | Group-based WL intervention;  Grounded on Self-Determination Theory; University setting | 12 months; no follow-up | 0, 12 months | MVPA change (7day-PAR): +110.2 ± 150.1 min/wk; *d* = 0.73  Lifestyle PA change (LPAI; Likert-type scale): *d* = 1.14 | Tested mediators:  Perceived autonomy (LCE), perceived competence (IMI), motivational regulations for PA (SRQ-E)  Significant mediators:  Intervention ↑ **MVPA** through a partial mediation of perceived need support, autonomy and competence satisfaction, and intrinsic motivation. Intervention ↑ **Lifestyle PA** by ↑ autonomy and competence satisfaction. All correlations between these variables and PA outcomes were significant, except external regulation. | SEM (SmartPLS software); Formal test of mediation, using MacKinnon’s approach  Correlations | Strong  (*EPHPP Tool* - adapted)  Moderate (*Rhodes Tool* - adapted) |
| Anderson-Bill et al., 2011[8] | RCT Post-hoc; 3 arms | 204 (34% men); Age n.d.; BMI > 25 kg/m^2^ | Internet-based health promotion intervention (GTH); Grounded on Social Cognitive Theory; Community setting | GTH: 3 months + 13 months follow-up;  GTH + church-based supports: 16 months; no follow-up | 0, 7, 16 months | 16-month weight change:  GTH: -1.4 ± 7.2 kg  GTH + Church: -3.1 ± 8.8 kg (Cohen’s *d* could not be calculated)  PA change (pedometer, self-reported walking): Data not reported | Tested mediators:  Exercise self-efficacy, outcome expectations, and self-regulation (HBS-PA)  Significant mediators:  Intervention ↑ exercise self-efficacy, which mediated ↑ in **PA** at 16m. This variable also mediated, albeit marginally, 16-month **WL**. | SEM (LISREL softaware); Formal test of mediation, using MacKinnon’s approach | Strong  (*EPHPP Tool* - adapted)  Moderate (*Rhodes Tool* - adapted) |
| **Authors** | **Study Design** | **Sample** | **Intervention** | | **Assessment Points** | **Outcomes** | **Mediators** | **Mediation Analysis** | **Study Quality ^1^** |
|  |  |  | Aim, rationale, setting/format | Length + Follow-up |  |  |  |  |  |
| Palmeira et al., 2009[9] | RCT post-hoc; 2 arms | 193 women; Age, 38.4 ± 6.7 yr; BMI, 31.1 ± 4.1 kg/m^2^ | Group-based WL intervention;  Grounded on Social Cognitive Theory; University setting | 12 months; no follow-up | 0, 12 months | 12-month weight change: -5.6 ± 6.8 % equivalent to ≈ 4.6 kg; *d* = -0.82 | Tested mediators:  Body dissatisfaction (BIA), body shape concerns (BSQ)  Significant mediators:  Intervention-induced ↓ in body dissatisfaction and body shape concerns mediated 12-month **weight changes.** These variables were also significant in correlational analyses. | P&H; simple mediations; Formal test of mediation, using Shrout & Bolger’s approach, as well as Baron & Kenny’s.  Correlations | Moderate  (*EPHPP Tool* - adapted)  Moderate (*Rhodes Tool* - adapted) |
| Coughlin et al., 2013 [10] | RCT; 3arms (Personal contact - PC; interactive technology - IT; self-directed group - SD) | 880 (38% men); Age, 55.9 ± 8.7 yr; BMI, 30.91 ± 4.7 kg/m^2^ | WLM intervention after a 6-month WL phase; Grounded on Social Cognitive Theory, University setting | 30 months; no follow-up | 6-month randomization (baseline) and 30 months | 30-month weight change  PC: -4.0 ± 5.3 kg; *d* = -0.76  IT: -5.2 ± 5.8 kg; *d* = -0.90  SD: -5.9 ± 6.3 kg; *d* = -0.94 | Tested mediators:  Self-weighing (WMSQ)  Significant mediators:  Personal contact intervention-induced ↑ in self-weighing frequency mediated 30-month **weight changes.** | Regressions and Ancovas; Formal test of mediation, using MacArthur’s and MacKinnon’s approach | Strong  (*EPHPP Tool* - adapted)  Moderate (*Rhodes Tool* - adapted) |

Notes: *d*, Cohen’s *d*; 7-d PAR, 7-day Physical Activity Recall; BAS-MBSRQ, Body Areas Satisfaction from the Multidimensional Body-Self Relations Questionnaire; BIA, Body Image Assessment; BSQ, Body Shape Questionnaire; BMI, body mass index; DBE, Decisional Balance for Exercise Scale; DEBQ, Dutch Eating Behavior Questionnaire; Dif, difference; EPBS, Exercise Perceived Barriers scale; EPHPP, Effective Public Health Practice Project; ESC, Exercise Stages of Change – short form ESE, Bandura’s Exercise Self-Efficacy Scale; ExSE, Exercise Self-Efficacy Scale; F/V, fruit/vegetable; HBS-PA, Health Beliefs Survey – Physical Activity section; IMI, Intrinsic Motivation Inventory; IPAQ, International Physical Activity Questionnaire; LCE, Locus of Causality for Exercise Scale; LPAI, Lifestyle Physical Activity Index; MVPA, moderate-vigorous physical activity; NCT, non-controlled trial; N.R., not reported; PA, physical activity; P&H, Preacher & Hayes mediation procedures; PMT-SE, Protection Motivation Theory Scale – Self-Efficacy Subscale; PSCS, Tennessee Physical Self-Concept Scale RCT, randomized controlled trial; RCT post-hoc, secondary analyses of an existing RCT for outcomes that were not planned originally; PSPP, Physical Self-Perception Profile Questionnaire; SEEBS, Self-Efficacy for Exercise Behaviors scale; SRQ-E, Exercise Self-Regulation Questionnaire; SSRS scale, Saelens’ Self-Regulatory Skill Usage Scale; SEM, structural equation modeling; TFEQ, Three-Factor Eating Questionnaire; WCC, Ways of Coping Checklist; WEL, Weight Efficacy Lifestyle Scale ; WL, weight loss; WLM, weight loss maintenance; WMSQ, Weight Management Strategies Questionnaire; WSC, Weight Stages of Change – short form; ↑, increased; ↓, decreased.^1^ General study quality was evaluated with an adapted version of the Effective Public Health Practice Project (EPHPP) tool; the quality of mediation studies was also evaluated with the Rhodes’ adapted version of the checklist tool developed specifically for mediator analyses by Lubans, Foster and Biddle (2008) [11]. ** This study, corresponding to reference [41] in the main manuscript, is based on the same intervention as the study referenced as [65] in that document. *** These three studies, references [1], [4], and [7] in this document (corresponding to references [36], [39], and [42] in the main manuscript), are based on the same intervention.

References

1. Silva MN, Markland D, Carraca EV, Vieira PN, Coutinho SR, Minderico CS, Matos MG, Sardinha LB, Teixeira PJ: **Exercise autonomous motivation predicts 3-yr weight loss in women.** *Med Sci Sports Exerc* 2011, **43:**728-737. (Reference 36 - Main manuscript)

2. Annesi JJ, Unruh JL, Marti CN, Gorjala S, Tennant G: **Effects of the chach approach intervention on adherence to exercise in obese women: Assessing mediation of social cognitive theory factors.** *Research Quarterly for Exercise and Sport* 2011, **82:**99-108. (Reference 37 - Main Manuscript)

3. Roesch SC, Norman GJ, Villodas F, Sallis JF, Patrick K: **Intervention-mediated effects for adult physical activity: A latent growth curve analysis.** *Social Scince and Medicine* 2010, **71:**494-501. (Reference 38 - Main Manuscript)

4. Teixeira PJ, Silva MN, Coutinho SR, Palmeira AL, Mata J, Vieira PN, Carraca EV, Santos TC, Sardinha LB: **Mediators of weight loss and weight loss maintenance in middle-aged women.** *Obesity (Silver Spring)* 2010, **18:**725-735. (Reference 39 - Main Manuscript)

5. Perri MG, Limacher MC, Durning PE, Janicke DM, Lutes LD, Bobroff LB, Dale MS, Daniels MJ, Radcliff TA, Martin AD: **Extended-Care Programs for Weight Management in Rural Communities: The Treatment of Obesity in Underserved Rural Settings (TOURS) Randomized Trial.** *Archives of Internal Medicine* 2008, **168:**2347-2354. (Reference 40 - Main Manuscript)

6. Burke V, Beilin LB, Cutt HE, Mansour J, Mori TA: **Moderators and mediators of behaviour change in a lifestyle program for treated hypertensives: a randomized controlled trial (ADAPT).** *Health Education Research* 2008, **23:**583–591. (Reference 41 - Main Manuscript)

7. Silva MN, Markland D, Vieira PN, Coutinho SR, Carraça EV, Palmeira AL, Minderico CS, Matos MG, Sardinha LB, Teixeira PJ: **Helping overweight women become more active: Need support and motivational regulations for different forms of physical activity.** *Psychology of Sport and Exercise* 2010, **11:**591-601. (Reference 42 - Main Manuscript)

8. Anderson-Bill ES, Winett RA, Wojcik JR, Williams DM: **Aging and the Social Cognitive Determinants of Physical Activity Behavior and Behavior Change: Evidence fromthe Guide to Health Trial.** *Journal of Aging Research* 2011, **(doi:10.4061/2011/505928)**. (Reference 43 - Main Manuscript)

9. Palmeira AL, Markland D, Silva MN, Branco TL, Martins SC, Minderico CS, Vieira PN, Barata JT, Serpa SO, Sardinha LB, Teixeira PJ: **Reciprocal effects among changes in weight, body image, and other psychological factors during behavioral obesity treatment: a mediation analysis.** *Int J Behav Nutr Phys Act* 2009, **6:**9. (Reference 44 - Main Manuscript)

10. Coughlin JW, Gullion CM, Brantley PJ, Stevens VJ, Bauck A, Champagne CM, Dalcin AT, Funk KL, Hollis JF, Jerome GJ, et al: **Behavioral mediators of treatment effects in the weight loss maintenance trial.** *Ann Behav Med* 2013, **46:**369-381. (Reference 45 - Main Manuscript)

11. Lubans DR, Foster C, Biddle S: **A review of mediators of behavior in interventions to promote physical activity among children and adolescents.** *Preventive Medicine* 2008, **47:**463-470.
