# Supplementary material for: Successful behavior change in obesity interventions in adults: a systematic review of self-regulation mediators
Source: BMC Med. 2015 Apr 16;13:84. doi: 10.1186/s12916-015-0323-6 (PMC4408562; doi:10.1186/s12916-015-0323-6)
Supplement: Additional file 3: — Characteristics of Included Studies Without Formal Mediation Analyses. [file 12916_2015_323_MOESM3_ESM.docx]

**Additional File 3**

**Table 3.1** Characteristics of included studies without formal mediation analyses

| **Authors** | **Study Design** | **Sample** | **Intervention** | | **Assessment Points** | **Outcomes** | **Predictors/Mediators** | **Statistical Analysis** | **Study Quality** |
| --- | --- | --- | --- | --- | --- | --- | --- | --- | --- |
|  |  |  | Aim, rationale, setting/format | Length + Follow-up |  |  |  |  |  |
| Lee et al., 2012[1] | NRCT (Randomized comparative trial);  2 arms | 310 women; Age, 43.5 ± 9.6 yr; BMI, 34.0 ± 9.7 kg/m^2^ | Group-cohesion based physical activity (PAI) OR dietary habits intervention (DHI);  Grounded on Group Dynamics Theory;  Community setting | 6 months; no follow-up | 0, 6 months | 6-month PA change (Accelerometry): PAI: -0.2 min/day (n.s.); DHI: +0.7 mon/day (n.s.)  6-month PA change (IPAQ): PAI: ≈ +452 Met.min.wk^-1^; Cohen’s *d* = 0.13; DHI: ≈ +1170 Met.min.wk^-1^; *d*=0.44  F/V intake change  PAI: ≈ +0.2 servings/day; *d*=0.07; DHI: ≈ +2.2 servings/day; *d*=0.83  Fat intake change  PAI: ≈ -1.5%; *d*= -0.44; DHI: ≈ -1.9%; *d*= -0.45 [National Cancer Institute F/V and fat screener] | Tested predictors/mediators:  Exercise self-efficacy (ESE); motivational readiness for PA (ESC) and for weight change (WSC)    Significant predictors/mediators:  PA intervention ↑ motivational readiness for PA (via ↑ task cohesion), which led to ↑ **self-reported PA** | SEM (LISREL software) | Moderate  (*EPHPP Tool* - adapted) |
| Annesi & Vaughn, 2011[2] | NCT;  1 arm | 88 women; Age, 41.9 ± 10.1 yr; BMI, 42.5 ± 6.2 kg/m^2^ | PA+ nutrition intervention;  Grounded on Social Cognitive Theory;  Exercise/Fitness club setting | 6 months; no follow-up | 0, 6 months | 6-month weight change: -2.4 ± 4.2 kg; *d* = -0.14 | Tested predictors/mediators:  Eating self-efficacy when negative emotions are present (WEL), self-regulatory skill usage for eating (SSRS scale)  Significant predictors/mediators:  All psychosocial variables improved during the intervention (*path a*). ↑ in eating self-efficacy and self-regulatory skill usage predicted **weight change** (*path b*). | T-tests, Multiple regressions | Weak  (*EPHPP Tool* - adapted) |
| Annesi & Gorjala, 2010[3] | NCT;  1 arm | 106 (23% men); Age, 43.5 ± 10.0 yr; BMI, 42.0 ± 6.0 kg/m^2^ | PA+ nutrition intervention;  Grounded on Social Cognitive Theory;  Exercise/Fitness club setting | 6 months; no follow-up | 0, 6 months | 6-month BMI change: -1.16 ± 1.73 kg/m^2^ equivalent to ≈ -3.4 kg; *d* = -0.19 | Tested predictors/mediators:  Eating self-efficacy (WEL), self-regulatory skill usage for eating (SSRS scale)  Significant predictors/mediators:  All psychosocial variables improved during the intervention (*path a*). ↑ in eating self-efficacy and self-regulatory skill usage predicted **BMI change** (*path b*). | T-tests, Regressions (univariate analyses) | Moderate  (*EPHPP Tool* - adapted) |
| **Authors** | **Study Design** | **Sample** | **Intervention** | | **Assessment Points** | **Outcomes** | **Mediators** | **Mediation Analysis** | **Study Quality** |
|  |  |  | Aim, rationale, setting/format | Length + Follow-up |  |  |  |  |  |
| Linde et al., 2006[4] | RCT; 2 arms | 349 (13% men); Age, 46.9 ± 8.6 yr; BMI, 33.9 ± 4.2 kg/m^2^ | Group-based WL intervention;  Grounded on Model of Behavior Change differentiating between initiation and maintenance [5];  University setting | 2 months (optimistic vs. balanced expectations); 4 months follow-up | 0, weekly during the 2-month intervention, monthly during follow-up | 6-month weight change: -2.9 ± 5.1 kg;  *d* = -0.57 | Tested predictors/mediators:  Eating and exercise self-efficacy (WEL adapted), frequency of self-weighing, frequency of calorie monitoring  Significant predictors/mediators:  Self-monitoring behaviors improved during treatment, but eating and exercise self-efficacy ↓ (*path a*). Frequency of self-weighing during active intervention positively predicted 6-month **weight change**, as did change in both self-monitoring behaviors post-intervention (*path b*). | GLM/Manovas | Moderate (*EPHPP Tool* - adapted) |
| Riebe et al.,2005[6] | RCT; 2 arms | 842 (52% men); Age, 42.6 ± 8.42 yr; BMI, 32.5 ± 3.8 kg/m^2^ | Group-based WL intervention + extended care;  Grounded on Transtheoretical Model (TTM); University setting | 6 months WL intervention + 6 months TTM-based (vs. control) extended care; 12 months follow-up | 0, 6, 12, 24 months | 24-month MVPA change: ≈ + 63 min/wk; *d* = 0.55  24-month changes: Caloric intake: ≈ -407 kcal; *d* = -0.72  Total fat intake: ≈ -0.7%; *d* = -0.09  Saturated fat intake: ≈ -0.8%; *d* = -0.26  F/V intake: ≈ -1.1 servings/day; *d* = -0.50 [3day Food Records] | Tested predictors/mediators:  Decisional balance (pros and cons) for reducing fat intake (DBDFR) and exercise (DBE), processes of change for dietary fat reduction (DFRPC) and exercise (PAPC), dietary self-efficacy (EatSE) and exercise self-efficacy (ExSE).  Significant predictors/mediators:  Self-efficacy, experiential and behavioral processes for PA and fat intake reduction ↓ from 6 to 24 months (*path a*). Significant time x **exercise** maintenance group interaction: those who stopped meeting guideline (150 min/wk), ↓ exercise self-efficacy (*path b*). There weren’t significant time x dietary fat maintenance group interactions. | Manovas | Weak  (*EPHPP Tool* - adapted) |
| Gallagher et al., 2006[7] | RCT; 4 arms | 165 women; Age, 37.6 ± 5.5 yr; BMI, 32.7 ± 4.2 kg/m^2^ | Group-based WL intervention;  Grounded on Social Cognitive Theory; University setting | 6 months (combinations of doses and intensities of PA); no follow-up | 0, 6 months | 6-month weight change: -8.7 ± 4.7 kg; *d* = -2.62*  6-month MVPA change (7d-PAR): +150.33 ± 314.24 min/wk; *d* = 0.48  * Authors did not present specific weight outcomes for each group | Tested predictors/mediators:  Exercise self-efficacy (ExSE), PA decisional balance for exercise - pros and cons (DBE), PA processes of change (PAPC), PA benefits and barriers (EOBHPA)  Significant predictors/mediators:  Exercise self-efficacy, PA psychological benefits, behavioral processes, increasing knowledge and increasing healthy opportunities ↑; expected barriers and caring about consequences to others ↓ (*path a*). **WL and PA** were associated with exercise self-efficacy, decisional balance, behavioral processes, and expected barriers (inversely; with PA, only time-related barriers) (*path b*). | Anovas, Correlations (Bivariate analyses) | Moderate  (*EPHPP Tool* - adapted) |
| **Authors** | **Study Design** | **Sample** | **Intervention** | | **Assessment Points** | **Outcomes** | **Mediators** | **Mediation Analysis** | **Study Quality** |
|  |  |  | Aim, rationale, setting/format | Length + Follow-up |  |  |  |  |  |
| Annesi & Marti, 2011[8] | NCT; 1 arm | 114 (23% men); Age, 43.3 ± 10.0 yr; BMI, 42.0 ± 6.0 kg/m^2^ | PA+ nutrition intervention;  Grounded on Social Cognitive Theory;  Exercise/Fitness club setting | 6 months; no follow-up | 0, 6 months | 6-month BMI change: -2.08 ± 1.47 kg/m^2^ equivalent to ≈ -6 kg; *d* = -0.34  6-month PA change (GLTEQ): *d* = 1.93 | Tested predictors/mediators:  Exercise self-efficacy (ExSE), physical self-concept (PSCS), body satisfaction (BAS-MBSRQ), self-regulatory skill usage for eating and for exercise (SSRS scale)  Significant predictors/mediators:  All psychosocial variables improved during the intervention (*path a*). ↑ self-regulation for PA was correlated and predicted **PA change and BMI reduction**; ↑ self-regulation for eating was correlated (but did not predict) **BMI change** (*path b*). Physical self-concept and body satisfaction correlated with PA and BMI change. Exercise self-efficacy correlated only with BMI change. | Correlations, Path analysis | Strong  (*EPHPP Tool* - adapted) |
| Annesi et al., 2011[9] | NCT; 1 arm | 183 (23% men); Age, 43.9 ± 9.9 yr; BMI, 42.0 ± 5.9 kg/m^2^ | WL/PA intervention;  Grounded on Social Cognitive Theory;  Exercise/Fitness club setting | 6 months; no follow-up | 0, 6 months | 6-month PA change (GLTEQ):  *d* = 0.87  6-month change in V/F intake: +0.6±1.6 servings/day; *d* = 0.27  [Servings per day in typical week and weekend day – FIQ] | Tested predictors/mediators:  Eating self-efficacy (WEL), exercise self-efficacy (ExSE), self-regulatory skill usage for eating and for exercise (SSRS scale)  Significant predictors/mediators:  Exercise self-efficacy and self-regulatory skills for PA correlated and predicted **PA**. Change in eating self-efficacy and self-regulatory skills for diet correlated and predicted **V/F intake**. | Regressions  Correlations | Moderate  (*EPHPP Tool* - adapted) |
| Annesi, 2011[10] | NCT; 1 arm | 137 (~25.6% men); Age, 43.6 ± 9.9 yr; BMI, 42.2 ± 6.6 kg/m^2^ | PA+ nutrition intervention;  Grounded on Social Cognitive Theory;  Exercise/Fitness club setting | 6 months; no follow-up | 0, 6 months | 6-month weight change: mean, ≈ -3.5 kg; *d* = 0.19 | Tested predictors/mediators:  Eating self-efficacy (total and negative emotion subscale of the WEL), self-regulatory skill usage for eating and for exercise (SSRS scale)  Significant predictors/mediators:  All eating-related psychosocial variables improved during the intervention (*path a*). Changes in all three variables positively predicted **weight changes** (*path b*). | T-tests, Multiple regressions | Weak  (*EPHPP Tool* - adapted) |
| **Authors** | **Study Design** | **Sample** | **Intervention** | | **Assessment Points** | **Outcomes** | **Mediators** | **Mediation Analysis** | **Study Quality** |
|  |  |  | Aim, rationale, setting/format | Length + Follow-up |  |  |  |  |  |
| Palmeira et al., 2010[11] | RCT; 3 arms | 142 women; Age, 38.3 ± 5.8 yr; BMI, 30.2 ± 3.7 kg/m^2^ | Group-based WL intervention;  Grounded on Social Cognitive Theory and LEARN manual; University setting | 4 months WL intervention; 12 months WM (meetings; meetings + exercise sessions; controls)  *Data collapsed for the analysis* | 0, 4, 16 months | 16-month weight change: -3.7 ± 3.9% equivalent to ≈ 3.0 ± 3.1 kg-, *d* = -0.95  4-16 month weight change (no significant changes) | Tested predictors/mediators:  Body image dissatisfaction (BIA), body shape concerns (BSQ), physical self-worth and body attractiveness (PSPP)  Significant predictors/mediators:  All psychosocial variables improved during the WL intervention (*path a*). 4-month changes in body attractiveness, body dissatisfaction, physical self-worth were positively correlated with **16-month weight change**. Only body dissatisfaction predicted **weight change** in regressions. Changes in body dissatisfaction were positively correlated with **weight maintenance** (*path b*). | T-tests, Correlations, and Multiple regressions | Moderate  (*EPHPP Tool* - adapted) |
| Teixeira et al., 2006[12] | RCT; 2 arms | 136 (23% men); Age, 48.1 ± 4.4 yr; BMI, 30.6 ± 5.6 kg/m^2^ | Group-based WL intervention;  Theory?; University setting | 4 months WL intervention; 12 months WLM (online contact; controls)  *Data collapsed for the analysis* | 0, 4, 16 months | 16-month weight change: mean, -4.6 kg (Cohen’s *d* could not be calculated)  4-16 month weight change: mean, -0.8 kg (Cohen’s *d* could not be calculated) | Tested predictors/mediators:  Eating restraint, disinhibition, and hunger (TFEQ), binge eating (BES), exercise self-efficacy (SEEBS), exercise barriers (EPBS), exercise motivation (IMI), body shape concerns (BSQ), physical self-worth and body attractiveness (PSPP)  Significant predictors/mediators:  All psychosocial variables improved during the WL intervention (**path a**). 4-month changes in all variables related to 16-month **weight change**. Changes in exercise self-efficacy, barriers, motivation, and physical self-worth were positively related to **weight** **maintenance**. Only exercise motivation was a significant predictor in regressions (interest-enjoyment subscale) (*path b*). | T-tests, Correlations, Multiple regressions | Moderate  (*EPHPP Tool* - adapted) |
| Annesi, 2007[13] | NCT; 1 arm | 52 women; Age, 45.7 ± 10.4 yr; BMI, 37.0 ± 4.4 kg/m^2^ | PA+ nutrition intervention;  Grounded on Social Cognitive Theory;  Exercise/Fitness club setting | 6 months; no follow-up | 0, 6 months | 6-month weight change: -5.3 ± 7.0 kg; *d* = -0.42 | Tested predictors/mediators:  Physical self-concept (PSCS), body satisfaction (BAS-MBSRQ)  Significant predictors/mediators:  All psychosocial variables improved during the intervention (*path a*). Changes in all variables were positively correlated with **weight changes** (*path b*). In regressions, body satisfaction and physical self-concept remained significant predictors. | T-tests, Correlations, Regressions | Moderate  (*EPHPP Tool* - adapted) |
| **Authors** | **Study Design** | **Sample** | **Intervention** | | **Assessment Points** | **Outcomes** | **Mediators** | **Mediation Analysis** | **Study Quality** |
|  |  |  | Aim, rationale, setting/format | Length + Follow-up |  |  |  |  |  |
| Moore et al., 2011[14] | RCT; 4 arms | 311 women; Age, 41.0 ± 6.0 yr; BMI n.d. | WL intervention; Theory?; University setting | 6 months (4 popular diets: Atkins, Zone, LEARN, Ornish) + 6 months follow-up | 0, 6, 12 months | 6-12 month weight regain  Atkins: +1.6 ± 3.2 kg; *d* = 0.50; Zone: +0.6 ± 3.5 kg; *d* = 0.17; LEARN: +0.7 ± 3.6 kg; *d* = 0.19; Ornish: +0.2 ± 3.7 kg; *d* = 0.05 | Tested predictors/mediators:  Outcome expectations/realizations regarding shape and appearance (OE-PA)  Significant predictors/mediators:  Baseline outcome expectations did not differ by group and did not predict 6-12-month weight regain. Positive 6-month realizations regarding shape/appearance were correlated and predicted less regain (controlling for initial outcome expectations and initial WL), only in the Atkins group. | Anovas, Correlations, Multiple regressions | Strong  (*EPHPP Tool* - adapted) |
| Haapala et al., 2009[15] | RCT; 2 arms | 125 (22.4% men); Age, 38 ± 4.7 yr; BMI, 30.5 ± 2.8 kg/m^2^ | Mobile-phone operated WL intervention; Grounded on Social Cognitive Theory and a Contingency Model based on Contact features | 12 months; no follow-up | 0, 3, 6, 9, 12 months | 12-month weight change: mean, ≈ -4.5 kg; *d* = -0.34 | Tested predictors/mediators:  Eating self-efficacy (Bandura´s EatSE - adapted), frequency of weight reporting  Significant predictors/mediators:  Overall significant ↓ in eating self-efficacy and weight reporting (*path a*), and these variables were correlated with **weight changes**. In regressions, ↑ eating self-efficacy predicted **weight change** (*path b*). | Anovas, Correlations, Multiple regressions | Strong  (*EPHPP Tool* - adapted) |
| Annesi & Whitaker, 2008[16] | RCT; 3 arms | 57 women; Age, 44.4 ± 10.3 yr; BMI, 43.8 ± 2.9 kg/m^2^ | PA intervention;  Grounded on Social Cognitive Theory;  Exercise/Fitness club setting | 6 months (PA (CA); PA plus nutrition education (CA/CH); controls); no follow-up | 0, 6 months | 6-month PA attendance  CA: 43.2 ± 30.5%  CA/CH: 51.0 ± 30.0% | Tested predictors/mediators:  Physical self-concept (PSCS), body satisfaction (BAS-MBSRQ), exercise self-efficacy (ExSE)  Significant predictors/mediators:  All psychosocial variables improved during the intervention (*path a*). Changes in body satisfaction positively predicted **PA attendance** (*path b*). | T-tests, Multiple regressions | Weak  (*EPHPP Tool* - adapted) |
| Annesi, 2012[17-18] | RCT; 2 arms | 430 (17.4% men); Age, 42.5 ± 10.0 yr; BMI, 41.7 ± 6.5 kg/m^2^ | WL intervention;  Grounded on Social Cognitive Theory;  Exercise/Fitness club setting | 6 months (PA plus nutrition education – NE; PA plus cognitive-behavioral methods to control eating – CBN); no follow-up | 0, 6 months | 6-month PA change (GLTEQ)  NE: *d* = 0.87  CBN: *d* = 1.07  6-month change in F/V intake  NE: + 0.5 ± 1.4 servings/day; *d* = 0.24  CBN: + 1.0 ± 1.6 servings/day; *d* = 0.50  [Servings/day in typical week and weekend day – FIQ] | Tested predictors/mediators:  Exercise self-efficacy (ExSE)  Eating self-efficacy (WEL), self-regulatory skill usage for eating and for exercise (SSRS scale)  Significant predictors/mediators:  All psychosocial variables improved during the intervention (*path a*). Changes in exercise self-efficacy, self-regulatory skills for PA predicted ↑ in **PA volume** (*path b*). Change in eating self-efficacy, self-regulatory skills to control eating predicted ↑ in **F/V intake** (*path b*). | Anovas, Multiple regressions | Strong  (*EPHPP Tool* - adapted) |
| **Authors** | **Study Design** | **Sample** | **Intervention** | | **Assessment Points** | **Outcomes** | **Mediators** | **Mediation Analysis** | **Study Quality** |
|  |  |  | Aim, rationale, setting/format | Length + Follow-up |  |  |  |  |  |
| Karhunen et al., 2012[18] | RCT; 2 arms | 82 (21% men); Age, 49.5 ± 9.3 yr; BMI, 34.2 ± 2.5 kg/m^2^ | Group-based WL+ maintenance intervention; Theory?; University setting | 2 months WL + 6 months WLM (high satiety foods; low satiety foods); no follow-up | 0, 2, 4, 8 months | 8-month weight change: mean, ≈ 12 kg (Cohen’s *d* could not be calculated) | Tested predictors/mediators:  Flexible, rigid, and total restraint, disinhibition, and hunger (TFEQ), restraint, emotional and external eating (DEBQ), binge eating (BES)  Significant predictors/mediators:  All psychosocial variables improved during the intervention (*path a*). Changes in total and flexible restraint, disinhibition, hunger, external eating, binge eating were positively associated with **weight maintenance**. After group adjustment, disinhibition and total restraint were no longer significant predictors (*path b*). | Linear mixed-effect modeling, correlations | Weak  (*EPHPP Tool* - adapted) |
| Wing et al., 2008[19] | RCT; 3 arms | 261 (18% men); Age, 51.2 ± 10.2 yr; BMI, 28.5 ± 4.8 kg/m^2^ | Weight maintenance intervention; Grounded on Self-Regulation Theory; Hospital setting | 18 months (internet; face-to-face; controls); no follow-up | 0, 6, 12, 18 months | Weight regain (changes in weight were not reported) | Tested predictors/mediators:  Restraint, disinhibition, and hunger (TFEQ), weighing frequency  Significant predictors/mediators:  Self-weighing and restraint (only in the face-to-face group) improved during the intervention, as previously reported (*path a*). Changes in psychosocial variables and self-weighing frequency were negatively associated with **weight regain** (*path b*). | Linear mixed-effect modeling | Moderate  (*EPHPP Tool* - adapted) |
| McGuire et al., 2001[20] | RCT; 3 arms | 1044 (21% men); Age, 35.2 ± 6.3 yr; BMI, 27.0 ± 5.7 kg/m^2^ | Weight gain prevention intervention; Theory?; Community setting | 36 months (education only; education + lottery incentive; controls); no follow-up | 0, 12, 24, 36 months | 36-month weight change: +1.8 ± 6.5 kg; *d* = 0.55  36-month PA change (PAH): -2.9 ± 30.5 metabolic units; *d* = -0.10  36-month change in dietary intake  Caloric intake: -176 ± 1091 kcal; *d* = -0.16  Fat intake: -1.4 ± 8.2%; *d* = -0.17  [Block Food Frequency Questionnaire] | Tested predictors/mediators:  Total, flexible and rigid restraint (TFEQ), self-weighing frequency  Significant predictors/mediators:  Self-weighing improved during the intervention, as previously reported (*path a*). Increases in these variables were related to positive changes in **caloric intake, fat intake, PA, and weight/BMI** (*path b*). | Regressions controlling for treatment group | Weak  (*EPHPP Tool* - adapted) |
| **Authors** | **Study Design** | **Sample** | **Intervention** | | **Assessment Points** | **Outcomes** | **Mediators** | **Mediation Analysis** | **Study Quality** |
|  |  |  | Aim, rationale, setting/format | Length + Follow-up |  |  |  |  |  |
| Burke et al., 2008[21] ** | RCT; 2 arms | 241 (~52% men); Age 40-70 yr; mean BMI ~30 kg/m^2^ | Intervention to reduce blood pressure; Grounded on Theory of Planned Behavior, Health Belief Model, Transtheoretical Model, Social Cognitive Theory, Decisional Balance; University setting | 4 months + 8 months follow-up | 0, 4, 12 months | 12-month weight change: Data not reported.  12-month MVPA (7day-PAR): Data not reported.  12-month change in saturated fat intake: Data not reported. [3day Food Records] | Tested predictors/mediators:  Exercise self-efficacy and diet self-efficacy (PMT-SE), beliefs about benefits of behavior change (created measure), coping mechanisms – consumption and external (WCC-revised); barriers for PA/Diet (created measure)  Significant predictors/mediators:  Beliefs about benefits of PA and diet ↑ during the 12-month period, while barriers for PA and external coping mechanisms ↓ (*path a*). Changes in dietary self-efficacy predicted change in **saturated fat intake**. Change in exercise self-efficacy predicted time spent in **PA**. Changes in dietary self-efficacy, beliefs about PA, and barriers for PA predicted **weight change** at 12 months (*path b*). | General linear models, Multiple regressions | Moderate  (*EPHPP Tool* - adapted) |
| Warziski et al., 2008[22] | RCT; 4 arms | 170 (11.8% men); Age, 44.1 ± 8.8 yr; BMI between 27-43 kg/m^2^ | WL intervention; Grounded on Social Cognitive Theory; University setting | 12 months (combination of yes/no preference, and a calorie and fat restricted OR a vegetarian diet); 6 months follow-up | 0, 6, 12, 18 months | 18-month weight change: -3.0±5.2% to  -6.1±7.7% equivalent to ≈ -3.3 to -5.7 kg; *d* = -0.23 | Tested predictors/mediators:  Eating self-efficacy (WEL)  Significant predictors/mediators:  Eating self-efficacy ↑ during the intervention, with no differences between groups (*path a*). Changes in self-efficacy for eating were positively associated with **weight loss** (*path b*). | Linear mixed model | Strong  (*EPHPP Tool* - adapted) |
| Annesi & Mareno, 2014 [23] | RCT; 2 arms | 144 (22% men); Age, 45.2 ± 9.2 yr; BMI, 40.7 ± 4.9 kg/m^2^ | WL intervention;  Grounded on Social Cognitive Theory;  Naturalistic exercise setting | 6 months (PA plus nutrition education; PA plus cognitive-behavioral methods to control eating); no follow-up | 0, 3, 6 months | 6-month PA change (LTEQ): *d* = 2.20  6-month change in F/V consumption: *d* = 0.59 [servings/day in typical week and weekend day – FIQ] | Tested predictors/mediators:  Eating self-efficacy (WEL), self-regulatory skill usage for eating (SSRS scale)  Significant predictors/mediators:  All psychosocial variables improved during the intervention (*path a*). Changes in eating self-efficacy and self-regulatory skills to control eating predicted ↑ in **F/V intake** and **PA** (*path b*). | Mixed model repeated-measures ANOVA, Multiple regression models | Moderate  (*EPHPP Tool* - adapted) |
| **Authors** | **Study Design** | **Sample** | **Intervention** | | **Assessment Points** | **Outcomes** | **Mediators** | **Mediation Analysis** | **Study Quality** |
|  |  |  | Aim, rationale, setting/format | Length + Follow-up |  |  |  |  |  |
| Annesi, 2013^[24]^ | RCT; 2 arms | 200 (19% men); Age, 44.3± 5.5 yr; BMI, 39.8 ± 4.1 kg/m^2^ | WL intervention;  Grounded on Social Cognitive Theory;  Exercise/Fitness club setting | 6 months (PA plus nutrition education - NE; PA plus cognitive-behavioral methods to control eating - CBN); no follow-up | 0, 3, 6 months | 6-month PA change (GLTEQ)  NE: *d* = 0.98  CBN: *d* = 1.28  6-month change in F/V consumption  NE: *d* = 0.24  CBN: *d* = 0.55  [Servings/day in typical week and weekend day – FIQ] | Tested predictors/mediators:  Exercise self-efficacy (ExSE)  Eating self-efficacy (WEL), self-regulatory skill usage for eating and for exercise (SSRS scale)  Significant predictors/mediators:  All psychosocial variables improved during the intervention (*path a*). Changes in self-regulatory skills for PA predicted ↑ in **PA** (*path b*). Change in eating self-efficacy, self-regulatory skills to manage eating predicted ↑ in **F/V intake** (*path b*). All correlations between these variables and the outcomes were significant. | Mixed model repeated-measures ANOVA, Multiple regression models  Correlations | Moderate  (*EPHPP Tool* - adapted) |
| Annesi & Porter, 2013[25] | RCT; 2 arms | 294 (17% men); Age, 43.0 ± 9.5 yr; BMI, 40.5 ± 4.1 kg/m^2^ | WL intervention;  Grounded on Social Cognitive Theory;  Exercise/Fitness club setting | 6 months (PA plus nutrition education - NE; PA plus cognitive-behavioral methods to control eating - CBN); no follow-up | 0, 6 months | 6-month PA change (GLTEQ)  NE: *d* = 1.90  CBN: *d* = 2.41  6-month change in F/V consumption  NE: *d* = 0.21  CBN: *d* = 0.57  [Servings/day in typical week and weekend day – FIQ] | Tested predictors/mediators:  Exercise self-efficacy (ExSE)  Eating self-efficacy (WEL), self-regulatory skill usage for eating and for exercise (SSRS scale)  Significant predictors/mediators:  All psychosocial variables improved during the intervention (*path a*). Changes in all predictors were correlated with ↑ **PA**; and all but exercise self-efficacy were correlated with ↑ **F/V intake** (*path b*). In regressions, changes in exercise self-efficacy and self-regulatory skills predicted ↑ in **PA**; and changes in eating self-efficacy and self-regulatory skills predicted ↑in **F/V intake** (*path b*). | Mixed model repeated-measures ANOVA, Correlations, Multiple regression models | Strong  (*EPHPP Tool* - adapted) |
| Fitzpatrick et al (2013) [26] | RCT; 3 arms  (only used pooled data from the 2 active arms in this study) | 501 (39 % male); Age, 50.0 ± 8.7 yr; BMI > 25 kg/m^2^ | Blood Pressure lifestyle intervention; Grounded on Social Cognitive Theory, Self-Applied  Behavior Modification Techniques, and the Transtheoretical Model; Clinical setting | 6 months (lifestyle intervention; lifestyle intervention + DASH diet); no follow-up reported in this study | 0, 6 months | 6-month weight change: -5.3 ± 5.7 kg  6-month change in total caloric intake (Kcal), percent energy from fat, from carbohydrates and from protein: Data not reported [24h dietary recalls].  6-month change in PA energy expended (7-d PAR): Data not reported | Tested predictors/mediators:  Diet self-monitoring (frequency of food intake log)  Exercise self-monitoring (recorded minutes of exercise)  Significant predictors/mediators:  Exercise and diet self-monitoring increased during the intervention (*path a*). Diet self-monitoring positively associated with **weight change** and negatively with **total fat intake**; no association with carbohydrate or protein intake. Exercise self-monitoring positively associated with **weight change**; no association with changes in PA energy expenditure. | SEM (MPlus software) | Moderate  (*EPHPP Tool* - adapted) |

Notes: *d*, Cohen’s *d*; 7-d PAR, 7-day Physical Activity Recall; Bandura´s EatSE, Eating Self-Efficacy Scale [27]; BAS-MBSRQ, Body Areas Satisfaction from the Multidimensional Body-Self Relations Questionnaire; BES, Binge Eating Scale; BIA, Body Image Assessment; BSQ, Body Shape Questionnaire; BMI, body mass index; DBDFR, Rossi’s Decisional Balance for Dietary Fat Reduction; DBE, Decisional Balance for Exercise Scale; DEBQ, Dutch Eating Behavior Questionnaire; DFRPC, Dietary Fat Reduction Processes of Change; Dif, difference; EatSE, Rossi’s Self-Efficacy for Resisting High-Fat Foods Scale; EOBHPA, Expected Outcomes and Barriers for Habitual Physical Activity Scale; EPBS, Exercise Perceived Barriers scale; EPHPP, Effective Public Health Practice Project; ESC, Exercise Stages of Change – short form; ExSE, Exercise Self-Efficacy Scale; FIQ, Food Intake Questionnaire; F/V, fruit/vegetable; GLTEQ, Godin Leisure-Time Exercise Questionnaire; IMI, Intrinsic Motivation Inventory; MVPA, moderate-vigorous physical activity; NCT, non-controlled trial; N.R., not reported; OE-PA , Outcome Expectations from Physical Activity Participation; PA, physical activity; PAPC, Physical Activity Processes of Change Questionnaire; PMT-SE, Protection Motivation Theory Scale – Self-Efficacy Subscale; PSCS, Tennessee Physical Self-Concept Scale RCT, randomized controlled trial; RCT post-hoc, secondary analyses of an existing RCT for outcomes that were not planned originally; PSPP, Physical Self-Perception Profile Questionnaire; SEEBS, Self-Efficacy for Exercise Behaviors scale; SSRS scale, Saelens’ Self-Regulatory Skill Usage Scale; SEM, structural equation modeling; TFEQ, Three-Factor Eating Questionnaire; WCC, Ways of Coping Checklist; WEL, Weight Efficacy Lifestyle Scale ; WL, weight loss; WLM, weight loss maintenance; ↑, increased; ↓, decreased.^1^ General study quality was evaluated with an adapted version of the Effective Public Health Practice Project (EPHPP) tool. ** This study, corresponding to reference [65] in the main manuscript, is based on the same intervention as the study referenced as [41] in that document.

**References**

1. Lee RE, O'Connor DP, Smith-Ray R, Mama SK, Medina AV, Reese-Smith JY, Banda JA, Layne CS, Brosnan M, Cubbin C, et al: **Mediating effects of group cohesion on physical activity and diet in women of color: health is power.** *Am J Health Promot* 2012, **26:**e116-125. (Reference 46 - Main manuscript)

2. Annesi JJ, Vaughn LL: **Relationship of Exercise Volume with Change in Depression and Its Association with Self-Efﬁcacy to Control Emotional Eating in Severely Obese Women.** *Advances in Preventive Medicine* 2011, **(doi:10.4061/2011/514271)**. (Reference 47 - Main manuscript)

3. Annesi JJ, Gorjala S: **Relationship of Exercise Program Participation with Weight Loss in Adults with Severe Obesity: Assessing Psychologically Based Mediators.** *Southern Medical Journal* 2010, **103:**1119-1123. (Reference 48 - Main manuscript)

4. Linde JA, Rothman AJ, Baldwin AS, Jeffery RW: **The Impact of Self-Efficacy on Behavior Change and Weight Change Among Overweight Participants in a Weight Loss Trial.** *Health Psychology* 2006, **25:**282-291. (Reference 49 - Main manuscript)

5. Rothman AJ: **Toward a theory-based analysis of behavioral maintenance.** *Health Psychol* 2000, **19:**64-69.

6. Riebe D, Blissmer B, Greene G, Caldwell M, Ruggiero L, Stillwell KM, Nigg CR: **Long-term maintenance of exercise and healthy eating behaviors in overweight adults.** *Preventive Medicine* 2005, **40:**769-778. (Reference 50 - Main manuscript)

7. Gallagher KI, Jakicic JM, Napolitano MA, Marcus BH: **Psychosocial Factors Related to Physical Activity and Weight Loss in Overweight Women.** *Medicine and Sciences in Sports and Exercise* 2006, **38:**971-980. (Reference 51 - Main manuscript)

8. Annesi JJ, Marti CN: **Path analysis of exercise treatment-induced changes in psychological factors leading to weight loss.** *Psychology and Health* 2011, **26:**1081–1098. (Reference 52 - Main manuscript)

9. Annesi JJ: **Self-Regulatory Skills Usage Strengthens the Relations of Self-Efficacy for Improved Eating, Exercise, and Weight in the Severely Obese: Toward an Explanatory Model.** *Behavioral Medicine* 2011, **37:**71-76. (Reference 53 - Main manuscript)

10. Annesi JJ: **Behaviorally Supported Exercise Predicts Weight Loss in Obese Adults Through Improvements in Mood, Self-Efficacy, and Self-Regulation, Rather Than by Caloric Expenditure.** *The Permanente Journal* 2011, **15:**23-27. (Reference 54 - Main manuscript)

11. Palmeira AL, Branco TL, Martins SC, Minderico CS, Silva MN, Vieira PN, Barata JT, Serpa SO, Sardinha LB, Teixeira PJ: **Change in body image and psychological well-being during behavioral obesity treatment: Associations with weight loss and maintenance.** *Body Image* 2010, **7:**187-193. (Reference 55 - Main manuscript)

12. Teixeira PJ, Going SB, Houtkooper LB, Cussler EC, Metcalfe LL, Blew RM, Sardinha LB, Lohman TG: **Exercise motivation, eating, and body image variables as predictors of weight control.** *Medicine and Sciences in Sports and Exercise* 2006, **38:**179-188. (Reference 56 - Main manuscript)

13. Annesi JJ: **Relations of Changes in Physical self-appraisal and Perceived energy with Weight Change in obese Women beginning a supported exercise and nutrition information program.** *Social Behavior and Personality* 2007, **35:**295-300. (Reference 57 - Main manuscript)

14. Moore SD, King AC, Kiernan M, Gardner CD: **Outcome expectations and realizations as predictors of weight regain among dieters.** *Eating Behaviors* 2011, **12:**60-63. (Reference 58 - Main manuscript)

15. Haapala I, Barengo NC, Biggs S, Surakka L, Manninen P: **Weight loss by mobile phone: a 1year effectiveness study.** *Public Health Nutrition* 2009, **12:**2382-2391. (Reference 59 - Main manuscript)

16. Annesi JJ, Whitaker AC: **Weight Loss and Psychologic Gain in Obese Women—Participants in a Supported Exercise Intervention.** *The Permanente Journal* 2008, **12:**36-45. (Reference 60 - Main manuscript)

17. Annesi JJ: **Supported Exercise Improves Controlled Eating and Weight through Its Effects on Psychosocial Factors: Extending a Systematic Research Program Toward Treatment Development.** *The Permanente Journal* 2012, **16:**7-18. (Reference 61 - Main manuscript)

18. Karhunen L, Lyly M, Lapveteläinen A, Kolehmainen M, Laaksonen DE, Lähteenmäki L, Poutanen K: **Psychobehavioural Factors AreMore Strongly Associated with SuccessfulWeightManagement Than Predetermined Satiety Effect or Other Characteristics of Diet.** *Journal of Obesity* 2012, **(doi:10.1155/2012/274068)**. (Reference 62 - Main manuscript)

19. Wing RR, Papandonatos G, Fava JL, Gorin AA, Phelan S, McCaffery J, Tate DF: **Maintaining Large Weight Losses: The Role of Behavioral and Psychological Factors.** *Journal of Consulting and Clinical Psychology* 2008, **76:**1015–1021. (Reference 63 - Main manuscript)

20. McGuire MT, Jeffery RW, French SA, Hannan PJ: **The relationship between restraint and weight and weight-related behaviors among individuals in a community weight gain prevention trial.** *International Journal of Obesity* 2001, **25:**574-580. (Reference 64 - Main manuscript)

21. Burke V, Mansour J, Mori TA, Beilin LB, Cutt HE, Wilson A: **Changes in cognitive measures associated with a lifestyle program for treated hypertensives: a randomized controlled trial (ADAPT).** *Health Education Research* 2008, **23:**202–217. (Reference 65 - Main manuscript)

22. Warziski MT, Sereika SM, Styn MA, Music E, Burke LE: **Changes in self-efﬁcacy and dietary adherence: the impact on weight loss in the PREFER study.** *Journal of Behavioral Medicine* 2008, **31:**81–92. (Reference 66 - Main manuscript)

23. Annesi JJ, Mareno N: **Temporal Aspects of Psychosocial Predictors of Increased Fruit and Vegetable Intake in Adults with Severe Obesity: Mediation by Physical Activity.** *J Community Health* 2014. (Reference 67 - Main manuscript)

24. Annesi J: **Effects of treatment differences on psychosocial predictors of exercise and improved eating in obese, middle-age adults.** *J Phys Act Health* 2013, **10:**1024-1031. (Reference 68 - Main manuscript)

25. Annesi JJ, Porter KJ: **Reciprocal effects of treatment-induced increases in exercise and improved eating, and their psychosocial correlates, in obese adults seeking weight loss: a field-based trial.** *Int J Behav Nutr Phys Act* 2013, **10:**133. (Reference 69 - Main manuscript)

26. Fitzpatrick SL, Bandeen-Roche K, Stevens VJ, Coughlin JW, Rubin RR, Brantley PJ, Funk KL, Svetkey LP, Jerome GJ, Dalcin A, et al: **Examining the Behavioral Processes through which Lifestyle Interventions Promote Weight Loss: Results from the PREMIER Trial.** *Obesity (Silver Spring)* 2013. (Reference 70 - Main manuscript)

27. Bandura A: *Self-efficacy: the exercise of control.* New York: W.H. Freeman and Company; 1997.
