# Supplementary material for: Successful behavior change in obesity interventions in adults: a systematic review of self-regulation mediators
Source: BMC Med. 2015 Apr 16;13:84. doi: 10.1186/s12916-015-0323-6 (PMC4408562; doi:10.1186/s12916-015-0323-6)
Supplement: Additional file 4: — Indirect effects’ estimates in studies with formal mediation analysis. [file 12916_2015_323_MOESM4_ESM.docx]

**Additional File 4**

**Table 7.1** Indirect effects’ estimates in studies with formal mediation analysis

| **Authors** | **Tested Mediators** | **Outcomes** | **Estimate** | **Sig.**  **(p-values; 95% CI)** | **Mediation Analysis** |
| --- | --- | --- | --- | --- | --- |
| Silva et al., 2011[1] | 12-month autonomous motivation for PA  12-month introjected motivation for PA  12-month external motivation for PA  24-month autonomous motivation for PA  24-month introjected motivation for PA  24-month external motivation for PA | 24-month MVPA  36-month Weight Change | 0.12  0.04  0.003  -0.10  0.01  -0.005 | p < 0.05  n.s.  n.s.  p < 0.05  n.s.  n.s.  (N=221) | SEM/SMARTPLS*  Estimates are a product of correlation coefficients (path a x path b) |
| Annesi et al., 2011[2] | Exercise self-efficacy  Physical self-concept  Body satisfaction | 6-month PA attendance | 1.58  2.67  1.25 | [-0.520; 5.660]  [0.280; 7.080]  [-1.610; 5.310]  (N=137) | Preacher & Hayes Macro  Estimates are unstandardized regression coefficients |
| Roesch et al, 2010[3] | Exercise self-efficacy  Self-regulatory skill use for PA | 12-month PA | 0.17  0.24 | [0.040; 0.310]  [0.110; 0.400]  (N=842) | SEM/MPlus  Estimates are unstandardized coefficients |
| Teixeira et al., 2010[4] | Flexible restraint  Emotional eating  Exercise perceived barriers  Flexible restraint  Exercise self-efficacy  Body dissatisfaction | 12-month weight change  24-month weight change | -0.08  -0.05  -0.04  -0.04  -0.07  -0.08 | [-0.133; -0.041]  [-0.103; -0.013]  [-0.090; 0.004]  [-0.100; -0.004]  [-0.141; -0.025]  [-0.155; -0.003]  (N=225) | Preacher & Hayes Macro  Estimates are standardized regression coefficients |
| Perri et al., 2008[5] | Self-monitoring | 18-month weight change | (estimates could not be calculated) | (N=234) | Linear regression analysis |
| Burke et al., 2010[6] | Exercise self-efficacy  Barriers for PA  Beliefs about benefits of behavior change  Coping mechanisms – consumption  Coping mechanisms – external  Diet self-efficacy  Barriers to prudent diet  Beliefs about benefits of behavior change  Coping mechanisms – consumption  Coping mechanisms – external | 12-month MVPA  12-month food/energy intake | 0.02  0.04  0.01  -0.001  -0.001  -0.05  -0.02  -0.02  0.002  0.003 | [-0.020; 0.078]  [-0.008; 0.118]  [-0.021; 0.042]  [-0.021; 0.026]  [-0.035; 0.030]  [-0.127; -0.005]  [-0.083; 0.027]  [-0.085; 0.009]  [-0.023; 0.036]  [-0.029; 0.037]  (N=241) | Preacher & Hayes Macro  Estimates are standardized regression coefficients |
| Silva et al, 2010[7] | Perceived autonomy  Perceived competence  Intrinsic motivation for PA  Identified motivation for PA  Introjected motivation for PA  External motivation for PA  Perceived autonomy  Perceived competence  Intrinsic motivation for PA  Identified motivation for PA  Introjected motivation for PA  External motivation for PA | 12-month MVPA  12-month Lifestyle PA | 0.08  0.08  0.17  0.16  0.08  -0.01  0.07  0.05  0.15  0.16  0.10  -0.005 | p < 0.05  p < 0.05  p < 0.05  n.s.  n.s.  n.s.  p < 0.05  p < 0.05  n.s.  n.s.  n.s.  n.s.  (N=239) | SEM/SMARTPLS*  Estimates are a product of correlation coefficients (path a x path b) |
| **Authors** | **Tested Mediators** | **Outcomes** | **Estimate** | **Sig.**  **(p-values; 95% CI)** | **Mediation Analysis** |
| Anderson-Bill et al., 2011[8] | Exercise self-efficacy  Outcome expectations – Negative  Outcome expectations – Positive  Self-regulation (goal setting and planning)  Exercise self-efficacy  Outcome expectations – Negative  Outcome expectations – Positive  Self-regulation (goal setting and planning) | 16-month weight change  16-month PA | -0.09  0.02  -0.03  -0.09  0.15  -0.02  -0.04  0.00 | p < 0.100  p > 0.100  n.s.  n.s.  p < 0.100  p > 0.100  n.s.  n.s.  (N=204) | SEM/LISREL  Estimates are standardized coefficients |
| Palmeira et al., 2009[9] | Body dissatisfaction  Body shape concerns | 12-month weight change | 0.17  0.07 | [0.090; 0.290]  [0.010; 0.140]  (N=193) | Preacher & Hayes Macro  Estimates are standardized regression coefficients |
| Coughlin et al., 2013 [10] | Self-weighing | 30-month weight change:  Personal contact vs. interactive technology;  Personal contact vs. self-directed | 0.07  0.53 | p = 0.010  p = 0.006  (N=880) | Logistic regressions; Ancovas  Estimates are derived from logistic models ran separately for each strategy |

Notes: * Serial mediations were performed in both these studies instead of simple mediations. Thus, specific estimates for each mediator involved in the significant paths were not available. Therefore, indirect effects were estimated based on the correlation coefficients provided for path a and path b in each case. Given that specific p-values were also not provided, we opted for using a conservative p-value (p<0.05) to indicate significant mediators in the serial mediation models.

References

1. Silva MN, Markland D, Carraca EV, Vieira PN, Coutinho SR, Minderico CS, Matos MG, Sardinha LB, Teixeira PJ: **Exercise autonomous motivation predicts 3-yr weight loss in women.** *Med Sci Sports Exerc* 2011, **43:**728-737. (Reference 36 - Main manuscript)

2. Annesi JJ, Unruh JL, Marti CN, Gorjala S, Tennant G: **Effects of the chach approach intervention on adherence to exercise in obese women: Assessing mediation of social cognitive theory factors.** *Research Quarterly for Exercise and Sport* 2011, **82:**99-108. (Reference 37 - Main Manuscript)

3. Roesch SC, Norman GJ, Villodas F, Sallis JF, Patrick K: **Intervention-mediated effects for adult physical activity: A latent growth curve analysis.** *Social Scince and Medicine* 2010, **71:**494-501. (Reference 38 - Main Manuscript)

4. Teixeira PJ, Silva MN, Coutinho SR, Palmeira AL, Mata J, Vieira PN, Carraca EV, Santos TC, Sardinha LB: **Mediators of weight loss and weight loss maintenance in middle-aged women.** *Obesity (Silver Spring)* 2010, **18:**725-735. (Reference 39 - Main Manuscript)

5. Perri MG, Limacher MC, Durning PE, Janicke DM, Lutes LD, Bobroff LB, Dale MS, Daniels MJ, Radcliff TA, Martin AD: **Extended-Care Programs for Weight Management in Rural Communities: The Treatment of Obesity in Underserved Rural Settings (TOURS) Randomized Trial.** *Archives of Internal Medicine* 2008, **168:**2347-2354. (Reference 40 - Main Manuscript)

6. Burke V, Beilin LB, Cutt HE, Mansour J, Mori TA: **Moderators and mediators of behaviour change in a lifestyle program for treated hypertensives: a randomized controlled trial (ADAPT).** *Health Education Research* 2008, **23:**583–591. (Reference 41 - Main Manuscript)

7. Silva MN, Markland D, Vieira PN, Coutinho SR, Carraça EV, Palmeira AL, Minderico CS, Matos MG, Sardinha LB, Teixeira PJ: **Helping overweight women become more active: Need support and motivational regulations for different forms of physical activity.** *Psychology of Sport and Exercise* 2010, **11:**591-601. (Reference 42 - Main Manuscript)

8. Anderson-Bill ES, Winett RA, Wojcik JR, Williams DM: **Aging and the Social Cognitive Determinants of Physical Activity Behavior and Behavior Change: Evidence fromthe Guide to Health Trial.** *Journal of Aging Research* 2011, **(doi:10.4061/2011/505928)**. (Reference 43 - Main Manuscript)

9. Palmeira AL, Markland D, Silva MN, Branco TL, Martins SC, Minderico CS, Vieira PN, Barata JT, Serpa SO, Sardinha LB, Teixeira PJ: **Reciprocal effects among changes in weight, body image, and other psychological factors during behavioral obesity treatment: a mediation analysis.** *Int J Behav Nutr Phys Act* 2009, **6:**9. (Reference 44 - Main Manuscript)

10. Coughlin JW, Gullion CM, Brantley PJ, Stevens VJ, Bauck A, Champagne CM, Dalcin AT, Funk KL, Hollis JF, Jerome GJ, et al: **Behavioral mediators of treatment effects in the weight loss maintenance trial.** *Ann Behav Med* 2013, **46:**369-381. (Reference 45 - Main Manuscript)
