# Supplementary material for: Successful behavior change in obesity interventions in adults: a systematic review of self-regulation mediators
Source: BMC Med. 2015 Apr 16;13:84. doi: 10.1186/s12916-015-0323-6 (PMC4408562; doi:10.1186/s12916-015-0323-6)
Supplement: Additional file 5: — EPHPP Quality Assessment Tool (adapted by the SPOTLIGHT Consortium). [file 12916_2015_323_MOESM5_ESM.docx]

**Additional File 5**

**EPHPP Quality Assessment Tool (adapted by the SPOTLIGHT Consortium)**

**STUDY DESIGN**

(**Q1**) The study design is:

- 1. Experimental
     1. Individual-randomised
     2. Group-randomised
     3. Non-randomised
  2. Observational
     1. Cross-sectional
     2. Longitudinal (also natural experiment or pre-post tests)
     3. Case-control
  3. Any other method (i.e. pre-post test without control group) or did not state method

(**Q2**) Was this an intervention study?

**Yes** – proceed

**No** – go to question 7

(**Q3**) Is the intervention of interest clearly described?

1. Yes
2. No
3. Not applicable (if using an existing database and referring to design article*)

(**Q4**) Were (groups of) subjects randomized into intervention groups?

1. Yes
2. No
3. Can’t tell
4. Not applicable (if using an existing database and referring to design article*)

(**Q5**) Was the intervention assignment concealed from participants and care givers until recruitment was completed?

1. Yes
2. No
3. Can’t tell
4. Not applicable (if using an existing database and referring to design article*)

(**Q6**) Was (were) the intervention or exposure status of participants concealed from the outcome assessors?

1. Yes
2. No
3. Can’t tell
4. Not applicable (if using an existing database and referring to design article*)

(**Q7**) Were power/sample size calculations conducted?

1. Yes, details of calculation provided
2. Yes, no details provided
3. Not reported or post hoc computation
4. Not applicable (if using an existing database and referring to design article*)

**Rating study design: Strong:** Q1 is 1

**Moderate:** Q1 is 2

**Weak:** Q1 is 3

**Rating blinding: Strong:** Q5 and Q6 are 1

**Moderate:** Q5 or Q6 is 1**; or** Q5 or Q6 are 3

**Weak:** Q5 and Q6 are 2; **or** Q5 and Q6 are 3

**No rating:** Q5 and Q6 are 4

**(No rate is given when study is not an intervention study)**

** If the study is using data from a large existing database such as HSE, NHANES, BRFSS, etc., often the authors refer to the design paper of the original study and no information in the present article is being described about power calculations, validity of tools, intervention description, etc.*

**REPRESENTATIVENESS (selection bias)**

(**Q8**) Is the spectrum of individuals selected to participate likely to be representative of the wider population who experience the intervention/exposure/situation?

1. Very likely
2. Somewhat likely
3. Not likely (selected group of users e.g., volunteers)
4. Can´t tell*
5. Not applicable*

(**Q9**) What percentage of the selected participants agreed to participate?

1. …………..%
2. Can’t tell
3. Not applicable

(**Q10**) Were inclusion/exclusion criteria specified and number of exclusions reported?

1. Criteria and number of exclusions reported
2. Criteria or number of exclusions not reported
3. Criteria and number not reported
4. Not applicable (if using an existing database and authors refer to design article)

**Rating: Strong:** Q8 is 1

**Moderate:** Q8 is 2

**Weak:** Q8 is 3 or 4

**No rating:** Q8 is 5

******* *Rate the representativeness of each study uniquely, according to each study specific context (community, specific group of the population, particular place, etc.).* *If a paper is using a large national dataset and refers to a design paper in their methods section, we answer Q8 with 5 (not applicable). However, if the authors used an existing database and do not refer to a design article, the rating should be 4 (can’t tell). As a result, if you have rated Q8 with 5 (not applicable), than it is not possible to give a rating for representativeness.*

**REPRESENTATIVENESS (withdrawals and drop-outs)**

(**Q11**) Were withdrawals and drop-outs reported in terms of numbers and reasons per group?

1. Numbers and reasons provided
2. Numbers but no reasons provided
3. Can’t tell (if longitudinal data)
4. Not applicable (if cross-sectional data or if using an existing database and authors refer to design article)

*If Q11 is 1 or 2, proceed to Q12. Otherwise, proceed to Q13.*

(**Q12**) What was the loss to follow-up (report the percentage completing the study and if it differs by groups, record the lowest)?

1. ⁮ …………..%
2. ⁮ Not provided
3. ⁮ Not applicable

**Rating: Strong:** Q11 is 1

**Moderate:** Q11 is 2

**Weak:** Q11 is 3

**No rating:** Q11 is 4

**CONFOUNDERS**

(**Q13#**) What confounders were the analyses adjusted for?

…………………………………………………………………………………………………...

…………………………………………………………………………………………………...

(**Q13**) Were analyses appropriately adjusted for confounders?

1. For most confounders
2. For some confounders
3. No or can’t tell

*The following are examples of confounders: race, sex, marital status/family, age, SES (income or class), education, health status, pre-intervention score on outcome measure. Rate the confounding as good if the authors took into account several factors (independent of whether they treated them as confounders, covariables, moderators or mediators). Consider as minimum for ‘most confounders’ controlling for age, gender, SES.*

*Considering the study design, were appropriate methods for controlling confounding variables and limiting potential biases used? Confounding can be addressed by appropriate use of randomization, restriction, matching, stratification, or multivariable methods. Sometimes use of a single method may be inadequate. Some biases can be limited by institution of data collection or study procedures that support validity of the study (e.g. training and/or blinding of interviewers or observers, interviewers and observers are different from interventions’ implementers etc). Example: if between-group differences persist after randomization or matching, statistical control should also have been used.*

**Rating: Strong:** Q13 is 1

**Moderate:** Q13 is 2

**Weak:** Q13 is 3

**DATA COLLECTION**

(**Q14**) Were validity and reliability of the data collection tools discussed?

- 1. Both validity and reliability were discussed
  2. Validity or reliability were discussed
  3. None of them were discussed
  4. A larger dataset was used and authors provided adequate information to find information on validity and reliability

**Rating: Strong:** Q14 is 1

**Moderate:** Q14 is 2

**Weak:** Q14 is 3

**No rating:** Q14 is 4

**DATA ANALYSIS**

(**Q15**) Were appropriate statistical analyses conducted (including correction for multiple tests where applicable*)?

1. a. Statistical methods were described, appropriate and comprehensive, and used a sophisticated approach

b. Statistical methods were described, appropriate and comprehensive, and used a simple approach

1. Statistical methods were described and less appropriate
2. No description of statistical methods or inappropriate methods

**Rating: Strong:** Q15 is 1

**Moderate:** Q15 is 2

**Weak:** Q15 is 3

** Consider statistical analyses to be appropriate if they account for confounding factors (so correlation analysis only is not enough). Do not punish papers for not correcting for multiple tests, if the rest of the analyses are appropriate.*

**REPORTING**

(**Q16**) Are the hypotheses/aims/objectives of the study clearly described?

1. Yes
2. No

(**Q17**) Are the main outcomes to be measured clearly described?

1. Yes
2. No

(**Q18**) Are the main findings clearly described?

1. Yes
2. No

(**Q19**) Have actual probability values been reported (i.e., p = .345 instead of p > .050; same goes for t-values, 95% CIs, etc.)?

1. Yes
2. No

**Rating: Strong:** Q16 and Q19 are 1

**Moderate:** Q16 or Q19 are 1

**Weak:** Q16 and Q19 are 2

Studies can have between six and eight component ratings. The overall rating for each study is determined by assessing the component ratings. **Strong** will be attributed to those with no WEAK ratings and at least four STRONG ratings; **Moderate** will be given to those with one WEAK rating or fewer than four STRONG ratings; **Weak** will be attributed to those with two or more WEAK ratings. (If only six ratings have been given, Strong will be attributed to those with no WEAK ratings and at least three STRONG ratings.) The final decision of both reviewers will be: strong, moderate, or weak.

**Source of the questions in the quality assessment tool**

Questions are derived from the following tools: Thomas, Black and Downs, CCDAN and Newcastle Ottawa. Below is an overview of the source of each question:

Study design

Study design

Were subjects randomized into intervention groups?

*Black and Downs (CCDAN)*

Was the randomized intervention assignment concealed from participants and care givers until recruitment was completed? *Black and Downs (CCDAN)(Thomas)*

Blinding

Was (were) the outcome assessor(s) aware of the intervention or exposure status of participants? *Thomas (Downs and Black)(Newcastle Ottawa)(CCDAN)*

Were the participants aware of the research question?

*Thomas (Downs and Black)*

Representativeness (selection bias)

**A**re the individuals selected to participate likely to be representative?

*Thomas (Black and Downs)(Newcastle Ottawa)*

What percentage of the selected participants agreed to participate?

*Thomas*

Were power/sample size calculations conducted?

*CCDAN (Downs and Black)*

Were inclusion/exclusion criteria specified and number of exclusions reported?

*CCDAN*

Representativeness (withdrawals and drop-outs)

Were withdrawals and drop-outs reported in terms of numbers and reasons per group?

*CCDAN (Thomas)(Downs and Black)*

What was the loss to follow-up/percentage completing the study? (If % differs by groups, record the lowest)

*Thomas*

Confounders

Were analyses appropriately adjusted for confounders?

*Thomas (Downs and Black) (Newcastle Ottawa)(CCDAN)*

Data collection

Were data collection tools shown to be valid and reliable?

*Thomas (Downs and Black) (CCDAN)*

Note: Because it might be difficult to assess whether there are valid and reliable tools for all kind of variables in the studies, we changed this question to ‘Were validity and reliability of the data collection tools discussed?’. We did this because it may be more important to know how the researchers handled the issue of the availability of valid/reliable tools.

Data analysis

Were appropriate statistical analyses conducted (including correction for multiple tests where applicable)? *CCDAN (Thomas) (Downs and Black) (Newcastle Ottawa)*

Reporting

Is the hypothesis/aim/objective of the study clearly described?

*Downs and Black (CCDAN)*

Are the main outcomes to be measured clearly described?

*Downs and Black (CCDAN)*

Are the main findings clearly described?

*Downs and Black (CCDAN)*

Have actual probability values been reported?

*Downs and Black*

Is the intervention of interest clearly described?

*Downs and Black (CCDAN)*

**References**

Downs SH, Black BN. (1998). The feasibility of creating a checklist for the assessment of the methodological quality both of randomised and non-randomised studies of health care interventions. *J Epidemiol Commun Health*;52:377–84.

Effective Public Health Practice Project. (1998). Quality Assessment Tool For Quantitative Studies. Hamilton, ON: Effective Public Health Practice Project.

Moher, D, Cook, DJ, Jadad, AR, Tugwell, P, Moher, M, Jones, A, et al. (1999). Assessing the quality of reports of randomised trials: Implications for the conduct of meta-analyses. *Health Technology Assessment, 3,* i–iv, 1–98.

Thomas BH, Ciliska D, Dobbins M, Micucci S. (2004). A process for systematically reviewing the literature: providing the research evidence for public health nursing interventions. . *Worldviews on Evidence-Based Nursing*, 1:176-184.

Wells GA, Shea B, O'Connell D, Peterson J, Welch V, Losos M, et al. The Newcastle-Ottawa Scale (NOS) for assessing the quality if nonrandomized studies in meta-analyses. http://www ohrica/programs/clinical_epidemiology/oxford htm 2009 [cited 2009 Oct 19];Available from: URL: http://www.ohri.ca/programs/clinical_epidemiology/oxford.htm

**Mediation Study Quality Checklist (adapted by Rhodes and Pfaeffli, 2010)**

**STUDY # RATER __________**

(**Q1**) Did the study cite/include a theoretical framework?

- 1. Yes (1)
  2. No (0)

(**Q2**) Were the study methods/procedures designed to influence mediating variables?

1. Yes (1)
2. No (0)

(**Q3**) Did the authors report conducting pilot studies to test mediation?

1. Yes (1)
2. No (0)

(**Q4**) Were the outcomes assessed with objective measures?

1. Yes (1)
2. No (0)

(**Q5**) Were the outcome measures reliable?

1. Yes (if needed, check study design papers) (1)
2. No or can’t tell (0)

(**Q6**) Were mediator measures reliable (i.e., Cronbach’s alpha)?

1. Yes (if needed, check study design papers) (1)
2. No or can’t tell (0)

(**Q7**) Was the study adequately powered to detect mediation (i.e., reports sample size calculations for this purpose)?

1. Yes (1)
2. No or can’t tell (0)

(**Q8**) Was the design an RCT?

1. Yes (1)
2. No (0)

(**Q9**) Were the outcomes controlled for baseline values?

1. Yes (1)
2. No or can’t tell (0)

(**Q10**) Were statistically appropriate/acceptable methods used to test mediation?

1. Yes (1)
2. No (0)

(**Q11**) Did the study ascertain whether changes in the mediating variables preceded changes in the outcome variables (i.e., longitudinal mediation analyses were conducted)?

1. Yes (1)
2. No (0)

The tool was created with similar scoring to the Cochrane Collaboration's instrument for assessing risk of bias and includes 11 questions answered with a yes (1) or no (0) format. **High** quality is considered with scores of 9 to 11, **moderate** quality was considered with scores of 5 to 8 and **low** quality was considered with scores of 0 to 4. The final decision of both reviewers will be: high, moderate, or low.

**References:**

Lubans DR, Foster C, Biddle S: A review of mediators of behavior in interventions to promote physical activity among children and adolescents. *Preventive Medicine* 2008, 47:463-470.

Rhodes RE, Pfaeffli LA: Review Mediators of physical activity behaviour change among adult non-clinical populations: a review update. *International Journal of Behavioral Nutrition and Physical Activity* 2010, 7:37-48.
