# Supplementary material for: Successful behavior change in obesity interventions in adults: a systematic review of self-regulation mediators
Source: BMC Med. 2015 Apr 16;13:84. doi: 10.1186/s12916-015-0323-6 (PMC4408562; doi:10.1186/s12916-015-0323-6)
Supplement: Additional file 6: — Complete results for weight change, physical activity and dietary behaviors. [file 12916_2015_323_MOESM6_ESM.docx]

**Additional File 6**

**Table 5.1. Complete results for short-term Weight Change (< 12 months) – 21 predictors**

| **Putative Mediators (change)** | **Formal Mediation Studies** | | | **All studies** | | |
| --- | --- | --- | --- | --- | --- | --- |
|  | **Nr of studies** | **Times tested** | **Effect, %** | **Nr of studies** | **Times tested** | **Effect, %** |
| **↑ Self-regulatory skill use** |  |  |  | **6** | **12** | **92^a^** |
| ↑ Self-monitoring/self-weighing |  |  |  | 2 | 4 | 100 |
| ↑ Self-regulatory skill use for PA |  |  |  | 2 | 3 | 100 |
| ↑ Self-regulatory skill use for eating |  |  |  | 4 | 5 | 80 |
| **↑ Self-efficacy/Barriers** |  |  |  | **6** | **9** | **67*^b^** |
| ↑ Exercise self-efficacy |  |  |  | 3 | 3 | 33* |
| ↓ Exercise perceived barriers |  |  |  | 1 | 1 | 100* |
| ↑ Eating self-efficacy |  |  |  | 4 | 5 | 80* |
| **↑ Body image/ Physical Self-Worth** |  |  |  | **2** | **6** | **67*** |
| ↑ Body satisfaction |  |  |  | 2 | 3 | 67* |
| ↑ Physical self-concept |  |  |  | 2 | 3 | 67* |
| **↓ Eating disinhibition** |  |  |  | **1** | **4** | **75*^c^** |
| ↓ Eating disinhibition |  |  |  | 1 | 1 | 0 |
| ↓ External eating |  |  |  | 1 | 1 | 100* |
| ↓ Emotional eating |  |  |  | 1 | 1 | 100* |
| ↓ Binge eating |  |  |  | 1 | 1 | 100* |
| **↑ Cognitive restraint** |  |  |  | **1** | **3** | **33*^a^** |
| ↑ Cognitive restraint (total) |  |  |  | 1 | 1 | 0 |
| ↑ Flexible restraint |  |  |  | 1 | 1 | 100* |
| ↑ Rigid restraint |  |  |  | 1 | 1 | 0 |
| **↑ Processes of change** |  |  |  | **1** | **2** | **50*** |
| ↑ Experiential processes of change |  |  |  | 1 | 1 | 0 |
| ↑ Behavioral processes of change |  |  |  | 1 | 1 | 100* |
| **↑ Decisional balance (pros/cons)** |  |  |  | **1** | **1** | **100*** |
| **↓ Perceived hunger** |  |  |  | **1** | **1** | **100*^c^** |
| **↑ Outcome expectations/Beliefs** |  |  |  | **1** | **1** | **0** |
| ↑ Beliefs about benefits of PA |  |  |  | 1 | 1 | 0 |

Notes: *Times tested*, refers to the number of times a variable was analyzed; *Effect, %,* refers to the number of times an effect was found and is expressed in percentage. Since mediation analyses were not conducted, results are organized according to the number of times each major category of variables was tested in the overall analyses, depicted in the fifth column, in descent order. * ≥ 50% of these effects are based on correlational analyses. ^a^ Tested twice in weak quality studies; ^b^ tested three times in weak quality studies; ^c^ tested in a weak quality study.

**Table 5.2. Complete results for long-term Weight Change (≥ 12 months) – 30 predictors**

| **Putative Mediators (change)** | **Formal Mediation Studies** | | | **All studies** | | |
| --- | --- | --- | --- | --- | --- | --- |
|  | **Nr of studies** | **Times tested** | **Effect, %** | **Nr of studies** | **Times tested** | **Effect, %** |
| **↓** **Controlled motivation for PA** | **1** | **4** | **0** | **1** | **8** | **0** |
| ↓ External regulation for PA | 1 | 2 | 0 | 1 | 4 | 0 |
| ↓ Introjected regulation for PA | 1 | 2 | 0 | 1 | 4 | 0 |
| **↑ Self-regulatory skill use** | **3** | **3** | **75** | **6** | **6** | **83^a^** |
| ↑ Self-monitoring/self-weighing | 2 | 2 | 100 | 5 | 5 | 100* |
| ↑ Goal setting and planning | 1 | 1 | 0 | 1 | 1 | 0 |
| **↑ Body image/Physical Self-Worth** | **2** | **3** | **100** | **4** | **34** | **62*** |
| ↓ Body shape concerns | 1 | 1 | 100 | 4 | 9 | 44* |
| ↓ Body dissatisfaction | 2 | 2 | 100 | 3 | 8 | 100* |
| ↑ Body attractiveness |  |  |  | 3 | 9 | 44* |
| ↑ Physical self-worth |  |  |  | 3 | 8 | 63* |
| **↑ Self-efficacy/Barriers** | **2** | **3** | **67** | **6** | **28** | **68*** |
| ↑ Exercise self-efficacy | 2 | 2 | 100 | 4 | 8 | 73* |
| ↓ Exercise perceived barriers | 1 | 1 | 0 | 3 | 8 | 50* |
| ↑ Eating self-efficacy |  |  |  | 4 | 6 | 83* |
| ↑ Diet perceived barriers |  |  |  | 1 | 1 | 0 |
| ↑ Perceived competence | 1 | 2 | 50 | 1 | 2 | 50 |
| **↑ Autonomous motivation for PA** | **1** | **2** | **100** | **2** | **8** | **100*** |
| ↑ Autonomous motivation for PA | 1 | 2 | 100 | 1 | 4 | 100* |
| ↑ Intrinsic motivation for PA |  | - | - | 2 | 64 | 100* |
| **↑ Flexible restraint** | **1** | **2** | **100** | **2** | **5** | **60** |
| **↑ Outcome expectations/Beliefs** | **1** | **2** | **0** | **3** | **6** | **50** |
| ↑ Positive outcome expectations | 1 | 1 | 0 | 1 | 1 | 0 |
| ↑ Positive outcome realizations (shape) |  |  |  | 1 | 2 | 100* |
| ↑ Beliefs about benefits of PA |  |  |  | 1 | 1 | 100 |
| ↑ Beliefs about benefits of prudent diet |  |  |  | 1 | 1 | 0 |
| ↓ Negative outcome expectations | 1 | 1 | 0 | 1 | 1 | 0 |
| **↓ Eating disinhibition** | **1** | **1** | **100** | **3** | **16** | **38*** |
| ↓ Eating disinhibition |  |  |  | 3 | 7 | 43* |
| ↓ External eating |  |  |  | 1 | 2 | 0 |
| ↓ Emotional eating | 1 | 1 | 100 | 1 | 3 | 67* |
| ↓ Binge eating |  |  |  | 1 | 4 | 25* |
| **↑ Cognitive restraint (total)** |  |  |  | **4** | **8** | **50*** |
| **↓ Perceived hunger** |  |  |  | **3** | **5** | **20*** |
| **↑ Rigid restraint** |  |  |  | **2** | **3** | **33*** |
| **↑ Coping mechanisms** |  |  |  | **1** | **2** | **0** |
| ↓ External coping mechanisms |  |  |  | 1 | 1 | 0 |
| ↓ Consumption coping mechanisms |  |  |  | 1 | 1 | 0 |

Notes: *Times tested*, refers to the number of times a variable was analyzed; *Effect, %,* refers to the number of times an effect was found and is expressed in percentage. Results are organized according to the number of times each major category of variables was tested in mediation analyses, depicted in the second column, in descent order. For categories not tested in mediation analyses, the fifth column (times tested in overall analyses) should be taken as the reference. * ≥ 50% of these effects are based on correlational analyses. ^a^ Tested once in a weak quality study.

**Table 5.3. Complete results for short-term Physical Activity (< 12 months) – 14 predictors**

| **Putative Mediators (change)** | **Formal Mediation Studies** | | | **All studies** | | |
| --- | --- | --- | --- | --- | --- | --- |
|  | **Nr of studies** | **Times tested** | **Effect, %** | **Nr of studies** | **Times tested** | **Effect, %** |
| **↑ Body image/Physical Self-Worth** | **1** | **2** | **50** | **3** | **6** | **67*^a^** |
| ↑ Body satisfaction | 1 | 1 | 0 | 3 | 3 | 67* |
| ↑ Physical self-concept | 1 | 1 | 100 | 3 | 3 | 67* |
| **↑ Self-efficacy/Barriers** | **1** | **1** | **0** | **10** | **15** | **67*** |
| ↑ Exercise self-efficacy | 1 | 1 | 0 | 9 | 12 | 58* |
| ↑ Eating self-efficacy |  |  |  | 2 | 2 | 100* |
| ↓ Exercise perceived barriers |  |  |  | 1 | 1 | 100* |
| **↑ Self-regulatory skill use** |  |  |  | **7** | **13** | **85*** |
| ↑ Self-monitoring/self-weighing |  |  |  | 1 | 1 | 0 |
| ↑ Self-regulatory skill use for PA |  |  |  | 5 | 9 | 100* |
| ↑ Self-regulatory skill use for Diet |  |  |  | 3 | 3 | 67* |
| **↑ Motivational readiness** |  |  |  | **1** | **2** | **50** |
| ↑ Motivational readiness for weight loss |  |  |  | 1 | 1 | 0 |
| ↑ Motivational readiness for PA |  |  |  | 1 | 1 | 100 |
| **↑ Processes of change** |  |  |  | **1** | **2** | **50*** |
| ↑ Experiential processes of change |  |  |  | 1 | 1 | 0 |
| ↑ Behavioral processes of change |  |  |  | 1 | 1 | 100* |
| **↑ Decisional balance (pros/cons)** |  |  |  | **1** | **1** | **100*** |
| **↑Outcome expectations/Beliefs** |  |  |  | **1** | **1** | **0** |
| ↑ Beliefs about benefits of PA |  |  |  | 1 | 1 | 0 |

Notes: Notes: *Times tested*, refers to the number of times a variable was analyzed; *Effect, %,* refers to the number of times an effect was found and is expressed in percentage. Results are organized according to the number of times each major category of variables was tested in mediation analyses, depicted in the second column, in descent order. For categories not tested in mediation analyses, the fifth column (times tested in overall analyses) should be taken as the reference. * ≥ 50% of these effects are based on correlational analyses. ^a^ Tested once in a weak quality study.

**Table 5.4. Complete results for long-term Physical Activity (≥ 12 months) – 23 predictors**

| **Putative Mediators (change)** | **Formal Mediation Studies** | | | **All studies** | | |
| --- | --- | --- | --- | --- | --- | --- |
|  | **Nr of studies** | **Times tested** | **Effect, %** | **Nr of studies** | **Times tested** | **Effect, %** |
| **↑ Autonomous motivation for PA** | **2** | **8** | **63** | **2** | **16** | **81*** |
| ↑ Autonomous motivation for PA | 1 | 2 | 100 | 1 | 4 | 100* |
| ↑ Intrinsic motivation for PA | 1 | 2^a^ | 50 | 1 | 4 | 75* |
| ↑ Identified regulation for PA | 1 | 2^a^ | 0 | 1 | 4 | 50* |
| ↑ Perceived autonomy | 1 | 2 | 100 | 1 | 4 | 100* |
| **↓** **Controlled motivation for PA** | **2** | **8** | **0** | **2** | **12** | **25*** |
| ↓ External regulation for PA | 2 | 4 | 0 | 2 | 6 | 0 |
| ↓ Introjected regulation for PA | 2 | 4 | 0 | 2 | 6 | 33* |
| **↑ Self-efficacy/Barriers** | **4** | **6** | **67^b^** | **6** | **12** | **75^c^** |
| ↑ Exercise self-efficacy | 3 | 3 | 67 | 5 | 6 | 83 |
| ↓ Exercise perceived barriers | 1 | 1 | 0 | 2 | 2 | 0 |
| ↑ Perceived competence | 1 | 2 | 100 | 1 | 4 | 100* |
| **↑ Outcome expectations/Beliefs** | **2** | **3** | **0** | **3** | **3** | **0** |
| ↑ Positive outcome expectations | 1 | 1 | 0 | 1 | 1 | 0 |
| ↑ Beliefs about benefits of PA | 1 | 1 | 0 | 2 | 2 | 0 |
| ↓ Negative outcome expectations | 1 | 1 | 0 | 1 | 1 | 0 |
| **↑ Self-regulatory skill use** | **2** | **2** | **50^b^** | **3** | **3** | **67^c^** |
| ↑ Self-monitoring/self-weighing |  |  |  | 1 | 1 | 100 |
| ↑ Self-regulatory skill use for PA | 1 | 1 | 100 | 1 | 2 | 100 |
| ↑ Goal setting and planning | 1 | 1 | 0 | 1 | 1 | 0 |
| **↑ Coping mechanisms** | **1** | **2** | 0 | **1** | **2** | 0 |
| ↓ External coping mechanisms | 1 | 1 | 0 | 1 | 1 | 0 |
| ↓ Consumption coping mechanisms | 1 | 1 | 0 | 1 | 1 | 0 |
| **↑ Decisional balance (pros/cons)** |  |  |  | **1** | **2** | **0** |
| **↑ Cognitive restraint** |  |  |  | **1** | **3** | **0** |
| ↑ Cognitive restraint (total) |  |  |  | 1 | 1 | 0 |
| ↑ Flexible restraint |  |  |  | 1 | 1 | 0 |
| ↑ Rigid restraint |  |  |  | 1 | 1 | 0 |
| **↑ Processes of change** |  |  |  | **1** | **2** | **0** |
| ↑ Experiential processes of change |  |  |  | 1 | 1 | 0 |
| ↑ Behavioral processes of change |  |  |  | 1 | 1 | 0 |

Notes: *Times tested*, refers to the number of times a variable was analyzed; *Effect, %,* refers to the number of times an effect was found and is expressed in percentage. Results are organized according to the number of times each major category of variables was tested in mediation analyses, depicted in the second column, in descent order. For categories not tested in mediation analyses, the fifth column (times tested in overall analyses) should be taken as the reference. * ≥ 50% of these effects are based on correlational analyses. ^a^ Due to multi-collinearity between intrinsic and identified motivation in the structural model (discussed in the original paper [ref 42]), only one test was considered as autonomous motivation in the manuscript’s Table 5, for each physical activity outcome; ^b^ tested once in a weak quality study; ^c^ tested twice in weak quality studies.

**Table 5.5. Complete results for sort-term Food/Energy Intake (< 12 months) – 8 predictors**

| **Putative Mediators (change)** | **Formal Mediation Studies** | | | **All studies** | | |
| --- | --- | --- | --- | --- | --- | --- |
|  | **Nr of studies** | **Times tested** | **Effect, %** | **Nr of studies** | **Times tested** | **Effect, %** |
| **↑ Self-efficacy/Barriers** |  |  |  | **6** | **12** | **75*** |
| ↑ Exercise self-efficacy |  |  |  | 3 | 4 | 25* |
| ↑ Eating self-efficacy |  |  |  | 5 | 8 | 100* |
| **↑ Self-regulatory skill use** |  |  |  | **5** | **12** | **75*** |
| ↑ Self-monitoring/self-weighing |  |  |  | 1 | 4 | 25 |
| ↑ Self-regulatory skill use for diet |  |  |  | 4 | 6 | 100* |
| ↑ Self-regulatory skill use for PA |  |  |  | 2 | 2 | 100* |
| **↑ Motivational readiness** |  |  |  | **1** | **4** | **0** |
| ↑ Motivational readiness for weight loss |  |  |  | 1 | 2 | 0 |
| ↑ Motivational readiness for PA |  |  |  | 1 | 2 | 0 |

Notes: *Times tested*, refers to the number of times a variable was analyzed; *Effect, %,* refers to the number of times an effect was found and is expressed in percentage. Since mediation analyses were not conducted, results are organized according to the number of times each major category of variables was tested in the overall analyses, depicted in the fifth column, in descent order. * ≥ 50% of these effects are based on correlational analyses.

**Table 5.6. Complete results for long-term Food/Energy Intake (≥ 12 months) – 16 predictors**

| **Putative Mediators (change)** | **Formal Mediation Studies** | | | **All studies** | | |
| --- | --- | --- | --- | --- | --- | --- |
|  | **Nr of studies** | **Times tested** | **Effect, %** | **Nr of studies** | **Times tested** | **Effect, %** |
| **↑ Self-efficacy/Barriers** | **1** | **2** | **50** | **3** | **8** | **25** |
| ↓ Diet perceived barriers | 1 | 1 | 0 | 2 | 2 | 0 |
| ↑ Eating self-efficacy | 1 | 1 | 100 | 3 | 6 | 33 |
| **↑ Coping mechanisms** | **1** | **2** | **0** | **2** | **4** | **0** |
| ↓ External coping mechanisms | 1 | 1 | 0 | 2 | 2 | 0 |
| ↓ Consumption coping mechanisms | 1 | 1 | 0 | 2 | 2 | 0 |
| **↑Outcome expectations/Beliefs** | **1** | **1** | **0** | **2** | **2** | **0** |
| ↑ Beliefs about benefits of prudent diet | 1 | 1 | 0 | 2 | 2 | 0 |
| **↑ Processes of change** |  |  |  | **1** | **8** | **0** |
| ↑ Experiential processes of change |  |  |  | 1 | 4 | 0 |
| ↑ Behavioral processes of change |  |  |  | 1 | 4 | 0 |
| **↑ Cognitive restraint** |  |  |  | **1** | **6** | **0** |
| ↑ Cognitive restraint (total) |  |  |  | 1 | 2 | 0 |
| ↑ Flexible restraint |  |  |  | 1 | 2 | 0 |
| ↑ Rigid restraint |  |  |  | 1 | 2 | 0 |
| **↑ Decisional balance (pros/cons)** |  |  |  | **1** | **4** | **0** |
| **↑ Self-regulatory skill use** |  |  |  | **2** | **2** | **100^a^** |
| ↑ Self-monitoring/self-weighing |  |  |  | 2 | 2 | 100 |

Notes: *Times tested*, refers to the number of times a variable was analyzed; *Effect, %,* refers to the number of times an effect was found and is expressed in percentage. Results are organized according to the number of times each major category of variables was tested in mediation analyses, depicted in the second column, in descent order. For categories not tested in mediation analyses, the fifth column (times tested in overall analyses) should be taken as the reference. ^a^ Tested twice in weak quality studies.
