# Supplementary material for: Successful behavior change in obesity interventions in adults: a systematic review of self-regulation mediators
Source: BMC Med. 2015 Apr 16;13:84. doi: 10.1186/s12916-015-0323-6 (PMC4408562; doi:10.1186/s12916-015-0323-6)
Supplement: Additional file 7: — Consensus Ratings of Methodological Study Quality. [file 12916_2015_323_MOESM7_ESM.docx]

**Additional File 7**

**Table 6.1** Consensus Ratings of Methodological Study Quality

| **Questions** | **1*** | **2** | **3** | **4** | **5** | **6** | **7** | **8** | **9** | **10** | **11** | **12** | **13** | **14** | **15** | **16** | **17** | **18** | **19** | **Design** | **Blinding** | **Selection bias** | **Drop-outs** | **Confoun**  **-ders** | **Data collection** | **Data**  **analysis** | **Report** | **Global rating** |
| --- | --- | --- | --- | --- | --- | --- | --- | --- | --- | --- | --- | --- | --- | --- | --- | --- | --- | --- | --- | --- | --- | --- | --- | --- | --- | --- | --- | --- |
| **Study** |  |  |  |  |  |  |  |  |  |  |  |  |  |  |  |  |  |  |  |  |  |  |  |  |  |  |  |  |
| Lee,  2012 | 1.i. | 1 | 1 | 1 | 1 | 3 | 3 | 3 | 76 | 2 | 2 | 61 | 1 | 1 | 1.a. | 1 | 1 | 1 | 1 | strong | moderate | weak | moderate | strong | strong | strong | strong | moderate |
| Silva,  2011 | 1.i. | 1 | 1 | 1 | 1 | 2 | 1 | 2 | 89 | 1 | 1 | 71 | 3 | 2 | 1.a. | 1 | 1 | 1 | 2 | strong | moderate | moderate | strong | strong | moderate | strong | moderate | strong |
| Annesi  2011a | 1.i. | 1 | 1 | 1 | 3 | 1 | 1 | 2 | - | 1 | 1 | 64 | 3 | 1 | 1.b. | 1 | 1 | 1 | 1 | strong | moderate | moderate | strong | strong | strong | strong | strong | strong |
| Annesi,  2011b | 1.iii | 1 | 1 | 2 | 4 | 4 | 3 | 2 | - | 2 | 3 | - | 3 | 1 | 1.b. | 1 | 1 | 1 | 1 | strong | no rating | moderate | weak | weak | strong | strong | strong | weak |
| Annesi,  2010 | 1.iii | 1 | 1 | 2 | 2 | 1 | 1 | 2 | - | 2 | 2 | 78 | 3 | 1 | 1.b. | 1 | 1 | 1 | 1 | strong | strong | moderate | moderate | weak | strong | strong | strong | moderate |
| Roesch,  2010 | 1.i. | 1 | 1 | 1 | 4 | 3 | 3 | 2 | - | 2 | 2 | - | 1 | 2 | 1.a. | 1 | 1 | 1 | 1 | strong | weak | moderate | weak | strong | moderate | strong | strong | weak |
| Teixeira  2010 | 1.i. | 1 | 1 | 1 | 1 | 2 | 1 | 2 | 89 | 1 | 1 | 72 | 1 | 2 | 1.b. | 1 | 1 | 1 | 1 | strong | moderate | moderate | strong | strong | moderate | strong | strong | strong |
| Perri,  2009 | 1.i. | 1 | 1 | 1 | 3 | 1 | 1 | 2 | 91 | 1 | 1 | 94 | 2 | 3 | 1.b. | 1 | 1 | 1 | 1 | strong | moderate | moderate | strong | moderate | no rating | strong | strong | strong |
| Burke,  2008 | 1.i. | 1 | 1 | 1 | 1 | 3 | 3 | 2 | - | 2 | 2 | 76 | 2 | 3 | 1.b. | 1 | 1 | 1 | 1 | strong | moderate | moderate | moderate | moderate | weak | strong | strong | moderate |
| Linde,  2006 | 1.i. | 1 | 1 | 1 | 2 | 2 | 1 | 2 | - | 2 | 1 | 60 | 1 | 1 | 1.b. | 1 | 1 | 1 | 1 | strong | weak | moderate | strong | strong | strong | strong | strong | moderate |
| Riebe,  2005 | 1.i. | 1 | 1 | 1 | 3 | 3 | 3 | 2 | 88 | 1 | 2 | 55 | 3 | 2 | 1.b. | 1 | 1 | 1 | 2 | strong | weak | moderate | moderate | weak | moderate | strong | moderate | weak |
| Silva,  2011 | 1.i. | 1 | 1 | 1 | 1 | 2 | 1 | 2 | 89 | 1 | 1 | 79 | 1 | 1 | 1.a. | 1 | 1 | 1 | 2 | strong | moderate | moderate | strong | strong | strong | strong | moderate | strong |
| Callagher,  2006 | 1.i. | 1 | 1 | 1 | 3 | 3 | 1 | 2 | 99 | 1 | 1 | 96 | 1 | 2 | 1.b. | 1 | 1 | 1 | 1 | strong | weak | moderate | strong | strong | moderate | strong | strong | moderate |
| Annesi,  2011c | 1.iii | 1 | 1 | 2 | 2 | 1 | 3 | 2 | - | 2 | 2 | 79 | 3 | 1 | 1.a. | 1 | 1 | 1 | 1 | strong | strong | moderate | moderate | strong | strong | strong | strong | strong |
| Annesi,  2011d | 1.iii | 1 | 1 | 2 | 2 | 3 | 1 | 2 | - | 2 | 2 | 79 | 2 | 1 | 1.b. | 1 | 1 | 1 | 2 | strong | weak | moderate | moderate | moderate | strong | strong | moderate | moderate |
| Anderson-Bill, 2011 | 1.ii | 1 | 1 | 1 | 1 | 3 | 3 | 2 | 61 | 2 | 2 | 73 | 1 | 2 | 1.a. | 1 | 1 | 1 | 1 | strong | moderate | moderate | moderate | strong | moderate | strong | strong | strong |
| Annesi  2011d | 1.iii | 1 | 1 | 2 | 2 | 3 | 1 | 2 | - | 2 | 3 | - | 1 | 1 | 1.b. | 1 | 1 | 1 | 1 | strong | weak | moderate | weak | strong | strong | strong | strong | weak |
| Palmeira  2010 | 1.i. | 1 | 1 | 1 | 3 | 2 | 3 | 2 | 96 | 2 | 2 | 67 | 1 | 2 | 1.b. | 1 | 1 | 1 | 1 | strong | weak | moderate | moderate | strong | moderate | strong | strong | moderate |
| Teixeira, 2006 | 1.i. | 1 | 1 | 1 | 3 | 3 | 1 | 2 | - | 1 | 2 | 86 | 1 | 2 | 1.b. | 1 | 1 | 1 | 1 | strong | weak | moderate | moderate | strong | moderate | strong | strong | moderate |

* Content of the items and scoring system of the Adapted EPHPP Quality Assessment Tool is available in Additional File 4

**Table 6.1** (cont.) Consensus Ratings of Methodological Study Quality

| **Questions** | **1*** | **2** | **3** | **4** | **5** | **6** | **7** | **8** | **9** | **10** | **11** | **12** | **13** | **14** | **15** | **16** | **17** | **18** | **19** | **Study design** | **Blinding** | **Selection bias** | **Drop-outs** | **Confoun-ders** | **Data**  **collection** | **Data Analysis** | **Report** | **Global rating** |
| --- | --- | --- | --- | --- | --- | --- | --- | --- | --- | --- | --- | --- | --- | --- | --- | --- | --- | --- | --- | --- | --- | --- | --- | --- | --- | --- | --- | --- |
| **Study** |  |  |  |  |  |  |  |  |  |  |  |  |  |  |  |  |  |  |  |  |  |  |  |  |  |  |  |  |
| Annesi, 2007 | 1.iii | 1 | 1 | 2 | 4 | 3 | 3 | 2 | - | 3 | 2 | 8 | 2 | 1 | 1.b. | 1 | 1 | 1 | 1 | strong | weak | moderate | moderate | moderate | strong | strong | strong | moderate |
| Moore,  2011 | 1.i. | 1 | 1 | 1 | 1 | 1 | 1 | 2 | 40 | 1 | 1 | 76 | 1 | 1 | 1.b. | 1 | 1 | 1 | 1 | strong | strong | moderate | strong | strong | strong | strong | strong | strong |
| Haapala,  2009 | 1.i. | 1 | 1 | 1 | 1 | 3 | 1 | 2 | 94 | 1 | 1 | 73 | 1 | 2 | 1.b. | 1 | 1 | 1 | 2 | strong | moderate | moderate | strong | strong | moderate | strong | moderate | strong |
| Annesi, 2008 | 1.i. | 1 | 1 | 1 | 3 | 3 | 2 | 2 | - | 2 | 2 | 38 | 3 | 1 | 1.b. | 1 | 1 | 1 | 1 | strong | weak | moderate | moderate | weak | strong | strong | strong | weak |
| Annesi, 2012 | 1.i. | 1 | 1 | 1 | 3 | 1 | 3 | 2 | - | 2 | 2 | 76 | 1 | 1 | 1.b. | 1 | 1 | 1 | 1 | strong | moderate | moderate | moderate | strong | strong | strong | strong | strong |
| Karhunen, 2012 | 1.i. | 1 | 1 | 1 | 3 | 3 | 3 | 2 | - | 2 | 2 | 83 | 2 | 3 | 1.b. | 1 | 1 | 1 | 1 | strong | weak | moderate | moderate | moderate | weak | strong | strong | weak |
| Wing,  2008 | 1.i. | 1 | 1 | 1 | 3 | 1 | 1 | 2 | 60 | 1 | 1 | 83 | 1 | 3 | 1.a. | 1 | 1 | 1 | 1 | strong | moderate | moderate | strong | strong | weak | strong | strong | moderate |
| Palmeira, 2009 | 1.i. | 1 | 1 | 1 | 3 | 2 | 3 | 2 | - | 2 | 2 | 86 | 3 | 2 | 1.b. | 1 | 1 | 1 | 1 | strong | weak | moderate | moderate | strong | moderate | strong | strong | moderate |
| McGuire, 2001 | 1.i. | 1 | 1 | 1 | 3 | 3 | 2 | 1 | - | 1 | 1 | 55 | 1 | 3 | 1.b. | 1 | 1 | 1 | 1 | strong | weak | strong | strong | strong | weak | strong | strong | weak |
| Burke,  2008 | 1.i. | 1 | 1 | 1 | 1 | 2 | 3 | 2 | - | 2 | 2 | 76 | 2 | 4 | 1.b. | 1 | 1 | 1 | 1 | strong | moderate | moderate | moderate | moderate | no rating | strong | strong | moderate |
| Warziski, 2008 | 1.i. | 1 | 1 | 1 | 1 | 1 | 3 | 2 | 67 | 1 | 1 | 78 | 2 | 1 | 1.a. | 1 | 1 | 1 | 1 | strong | strong | moderate | strong | moderate | strong | strong | strong | strong |
| Annesi, 2014 | 1.i. | 1 | 1 | 1 | 1 | 1 | 1 | 2 | 94 | 3 | 3 | - | 2 | 1 | 1.b | 1 | 1 | 1 | 1 | strong | strong | moderate | weak | moderate | strong | strong | strong | moderate |
| Annesi, 2013a | 1.i. | 1 | 1 | 1 | 1 | 1 | 1 | 2 | 97 | 3 | 3 | - | 2 | 1 | 1.b | 1 | 1 | 1 | 1 | strong | strong | moderate | weak | moderate | strong | strong | strong | moderate |
| Annesi 2013b | 1.i. | 1 | 1 | 1 | 1 | 1 | 1 | 2 | 97 | 3 | 1 | 80 | 2 | 1 | 1.b. | 1 | 1 | 1 | 1 | strong | strong | moderate | strong | moderate | strong | strong | strong | strong |
| Fitzpatrick  2013 | 1.i. | 1 | 1 | 1 | 1 | 1 | 1 | 2 | 78 | 1 | 2 | 88 | 1 | 3 | 1.a. | 1 | 1 | 1 | 1 | strong | strong | moderate | Moderate | strong | weak | strong | strong | moderate |
| Caughlin, 2013 | 1.ii. | 1 | 1 | 1 | 4 | 3 | 1 | 2 | 76 | 1 | 1 | 85 | 1 | 2 | 1.a. | 1 | 1 | 1 | 1 | strong | moderate | moderate | strong | strong | moderate | strong | strong | strong |

* Content of the items and scoring system of the Adapted EPHPP Quality Assessment Tool is available in Additional File 4

| **Questions** | **1*** | **2** | **3** | **4** | **5** | **6** | **7** | **8** | **9** | **10** | **11** | **Total rating** |
| --- | --- | --- | --- | --- | --- | --- | --- | --- | --- | --- | --- | --- |
| **Study** |  |  |  |  |  |  |  |  |  |  |  |  |
| Silva, 2011 | Yes | Yes | No | No | Yes | Yes | Yes | Yes | No or can't tell | Yes | Yes | Moderate quality |
| Annesi, 2011 | Yes | Yes | No | Yes | Yes | Yes | No or can't tell | Yes | No or can't tell | Yes | Yes | Moderate quality |
| Roesch, 2010 | Yes | Yes | No | No | Yes | Yes | No or can't tell | Yes | Yes | Yes | Yes | Moderate quality |
| Teixeira, 2010 | Yes | No | No | Yes | Yes | Yes | No or can't tell | Yes | Yes | Yes | Yes | Moderate quality |
| Perri, 2009 | Yes | Yes | No | Yes | Yes | Yes | No or can't tell | Yes | No or can't tell | Yes | Yes | Moderate quality |
| Burke, 2008 | Yes | Yes | No | No | Yes | Yes | No or can't tell | Yes | Yes | Yes | No | Moderate quality |
| Silva, 2010 | Yes | Yes | No | No | Yes | Yes | Yes | Yes | No or can't tell | Yes | No | Moderate quality |
| Anderson-Bill, 2011 | Yes | No | No | Yes | Yes | Yes | No or can't tell | Yes | Yes | Yes | Yes | Moderate quality |
| Palmeira, 2009 | Yes | No | No | Yes | Yes | Yes | No or can't tell | Yes | Yes | Yes | No | Moderate quality |
| Coughlin, 2013 | Yes | Yes | No | No | Yes | Yes | No or can't tell | Yes | Yes | Yes | Yes | Moderate quality |

**Table 6.2** Consensus Ratings of Mediation Study Quality

* Content of the items and scoring system of the Mediation Study Quality Checklist is available in Additional File 4
